# Sex biased expression of hormone related genes at early stage of sex differentiation in papaya flowers

**DOI:** 10.1038/s41438-021-00581-4

**Published:** 2021-07-01

**Authors:** Juan Liu, Li-Yu Chen, Ping Zhou, Zhenyang Liao, Hai Lin, Qingyi Yu, Ray Ming

**Affiliations:** 1 FAFU and UIUC Joint Center for Genomics and Biotechnology, Key Laboratory of Genetics, Breeding and Multiple Utilization of Crops, Ministry of Education; Fujian Provincial Key Laboratory of Haixia Applied Plant Systems Biology; College of Life Science, Fujian Agriculture and Forestry University, Fuzhou, 350002 Fujian China; 2 Key Laboratory of Plant Germplasm Enhancement and Specialty Agriculture, Chinese Academy of Sciences, Wuhan Botanical Garden, Wuhan, 430074 China; 3 Texas A&M AgriLife Research Center at Dallas, Texas A&M University System, Dallas, TX 75252 USA; 4 Department of Plant Pathology & Microbiology, Texas A&M University, College Station, TX 77843 USA; 5 Department of Plant Biology, School of Integrative Biology, University of Illinois at Urbana-Champaign, Urbana, IL 61801 USA

**Keywords:** Plant molecular biology, Plant hormones, Plant evolution

## Abstract

Sex types of papaya are controlled by a pair of nascent sex chromosomes, but molecular genetic mechanisms of sex determination and sex differentiation in papaya are still unclear. We performed comparative analysis of transcriptomic profiles of male and female floral buds at the early development stage before the initiation of reproductive organ primordia at which there is no morphological difference between male and female flowers. A total of 1734 differentially expressed genes (DEGs) were identified, of which 923 showed female-biased expression and 811 showed male-biased expression. Functional annotation revealed that genes related to plant hormone biosynthesis and signaling pathways, especially in abscisic acid and auxin pathways, were overrepresented in the DEGs. Transcription factor binding motifs, such as MYB2, GAMYB, and AP2/EREBP, were enriched in the promoters of the hormone-related DEGs, and transcription factors with those motifs also exhibited differential expression between sex types. Among these DEGs, we also identified 11 genes in the non-recombining region of the papaya sex chromosomes and 9 genes involved in stamen and carpel development. Our results suggested that sex differentiation in papaya may be regulated by multiple layers of regulation and coordination and involved transcriptional, epigenetic, and phytohormone regulation. Hormones, especially ABA and auxin, transcription factors, and genes in the non-recombination region of the sex chromosome could be involved in this process. Our findings may facilitate the elucidation of signal transduction and gene interaction in sex differentiation of unisexual flowers in papaya.

## Introduction

Unisexuality is one of the important evolutionary transitions in sexual reproductive systems of flowering plants. Unisexual flowers resulted from developmental arrest of one whorl of reproductive organs^1^. Based on the developmental mechanisms that lead to unisexual flowers, two categories of unisexual flowers were defined. Type I flowers are bisexual at initiation and become unisexual by the termination of development in one whorl of reproductive organs. Type II flowers are unisexual from inception and sex differentiation occurs before the initiation of female or male organ primordia. Four stages of sexual organ development are defined by summarizing unisexual flower development in different species, before initiation of stamen or carpel primordia, early in stamen or carpel development, pre-meiosis, and post-meiosis. Developmental arrest of reproductive organs could occur at any of the four stages^2^.

Papaya is a trioecious species with male, female, and hermaphrodite flowers on different plants. Recent studies suggested that hermaphrodite papaya was derived from sex reversal of male papaya during domestication^3^. Sex type of papaya is controlled by a pair of nascent sex chromosomes, XX for females, XY for males, and XY^h^ for hermaphrodites^4,5^. Typical papaya male flowers have a trace of aborted pistil while female flowers are entirely devoid of stamens. Papaya male flower belongs to the type I unisexual flower and its carpel development is arrested after the initiation of carpel primordia. Papaya female flower belongs to the type II unisexual flower, in which only female organ primordia is initiated^6^.

Studies in hermaphrodite flowers of model plant species led to the formulation of the ABCE model of floral organ identity. The B class genes combined with the C and E class genes determine the identity of stamens, while the C class genes combined with the E class genes specify carpel identity^7^. Genetic controls of type II flowers are likely among the genes involved in the short developmental process from floral commitment to reproductive organ identity^2^. In contrast, type I flowers can be result of mutations in a wide range of developmental processes. It was reported that the B and C class genes regulated the sex differentiation in dioecious species *Thalictrum dioicum* and *Spinacia oleracea*^8,9^. However, due to the diversity of stages and processes of developmental arrest in the type I flowers^2^, it is difficult to identify the sex determination genes due to the involvement of a wide range of developmental processes from initiation to maturation of reproductive organs.

Besides genetic factors, hormones and environmental factors can also change sex differentiation. Sex differentiation is regulated by the interaction of genetic factors, plant hormones, and environmental cues^4,10^. Environmental changes usually lead to fluctuation of endogenous hormone level and subsequently change the genetic regulation network that regulates flower development^11^. Several sex-differentiating genes have been identified to be involved in hormone biosynthesis or signaling pathway. Maize *tasselseed* mutants exhibited failure in abortion of gynoecium in male flowers (tassel)^12^. *Tasselseed 1* was cloned and annotated as a *lipoxygenase*, which is involved in jasmonate biosynthesis^13^. *Tasselseed 2*, encodes a short-chain alcohol dehydrogenase that probably targets GA for its degradation^14^. In melon, three sex determination genes have been cloned and two of them encoded enzymes involved in ethylene biosynthesis^15–17^.

With the progress of high-throughput sequencing, transcriptomic profiles are used to uncover the molecular processes regulating unisexual flowers in dioecious or monoecious species^18,19^. Sex-specifically or differentially expressed TFs, sex-linked genes and genes involved in hormone biosynthesis or signaling pathway have been analyzed to uncover the molecular mechanism of sex determination or differentiation^20^. In papaya, the sex-determining region of the Y chromosome and its corresponding region on the X chromosome have been fully sequenced^21^. Although comparative analysis of gene expression in flower tissues between male, female, and hermaphrodite plants have been reported using high-throughput SuperSAGE^22^, flower samples used in this study were at late stages of flower development, at which sex differentiation is completed and key genes that are involved in sex determination might be no longer expressed. In this study, we collected floral buds from male and female trees before the initiation of reproductive organ primordia and performed comparative analysis of transcriptomic profiling across the sex differentiation process. This study provides important information for understanding the molecular mechanism of sex differentiation in papaya.

## Materials and methods

### Plant materials and morphological observation of floral buds

Dioecious papaya cultivar ‘Zhong Huang’ was planted in the greenhouse of Fujian Agriculture and Forest University. Floral buds smaller than 1.0 mm were collected from male and female trees and stored in RNAlater (Sigma-Aldrich) to avoid RNA degeneration. At this stage, sepal and petal primordia have emerged, but reproductive organ primordia have not been initiated yet. The flowers were carefully screened under a microscope, only those with no visible stamens or carpels were used in this study. Paraffin sections were conducted following the protocol described in ref. ^23^ to further confirm developmental stages of male and female floral buds.

### RNA isolation and Illumina sequencing

The papaya male and female floral buds were used to isolate total mRNA after removing the bracts. Each sample has three biological replicates, and each replicate contains about 10 floral buds. Total RNAs were extracted with Tripure reagent following the manufacturer’s protocol. The quality and quantity of RNA samples were determined using a Nanodrop ND-2000 Spectrophotometer and a bioanalyser 2100 with RNA Nanochip (Agilent, Palo Alto, CA, USA). Approximately 1 μg of total RNA was used for library construction using Illumina TruSseq prep kit. Paired-end sequencing was performed on an Illumina HiSseq 2500. Sequencing data are available in NCBI database (PRJNA532376).

### Gene expression analysis

Clean reads were mapped to the papaya reference genome using Tophat2. Transcripts were then assembled by Cufflinks, and the FPKM (fragments per kilobase of exon per million fragments mapped) values of genes and differentially expressed genes were identified by Cuffdiff^24^.

### Functional annotation

The AgriGO online toolkit was used to perform GO enrichment analysis^25^. KOBAS web server was used for KEGG metabolic pathway annotation^26^. Hormone-related genes and transcription factors were downloaded from Arabidopsis Hormone database 2.0^27^ and PlantTFDB v3.0^28^. Those genes were used as query to identify homolog genes in papaya by blastp with *e*-value cutoff of 1e-5.

### Transcription factor binding site analysis

Multiple Em for Motif Elicitation (MEME) website suite was employed for functional analysis of transcription factor binding sites^29^. 2 kb upstream sequences of all hormone-related genes were used to identify the known transcriptional binding motifs by MEME program, Analysis of Motif Enrichment (AME). PROMO was used to predict potential experimentally proved transcription factor binding sites in DNA sequences within a dissimilarity margin less or equal than 15%^30^.

### Quantitative real-time PCR (qPCR) analysis

Eighteen genes were selected to validate their expression patterns. qPCR primers were designed by prime primer 3. Total RNA from male and female floral buds were reverse-transcripted into cDNA using Takara kit (PrimeScript™ RT reagent Kit with gDNA Eraser). Real-time qPCR was performed using TransStart ^®^ Top Green qPCR SuperMix (Transgen biotech) and analyzed on a BIORAD CFXP6 Real-Time System. Ubiquitin was used for normalization, and the expression ratio was calculated by the 2^−△△Ct^ formula.

## Results

### Morphological observation and dissecting samples for transcriptomic analysis

**Fig. 1:**
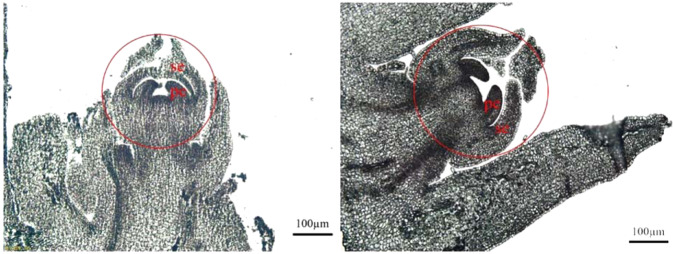
The paraffin longitudinal section of floral buds used in this study. Male (right) and Female (left) buds at petal primordium stage (red circled) before sexual differentiation were collected for RNA isolation. Se sepal, pe petal

Papaya floral meristems were initiated in the axils of bract that located on the side of broadened leaf bases in both male and female plants. At the early stage of floral development, five sepal primordia initiate following by five petal primordia (Fig. 1). At this stage, there is no obvious morphological difference between male and female flowers. When stamen primordia initiate in male flower, there are no traces of corresponding primordia observed in female flower. Besides, the pistils arisen in male flowers are degenerated into a spear-like structure after a brief time of development^6^. Therefore, floral buds before sexual differentiation, M0 for male floral buds and F0 for female floral buds were used for transcriptomic analysis to uncover the molecular regulatory mechanisms of unisexual flower development in papaya. We collected floral buds at stage 0 from papaya male and female trees in spring. After confirming the developmental stages under microscope, we dissected the center tissue within bracts for RNA sequencing.

### Male and female floral buds at stage 0 share similar transcriptomic profiles

**Fig. 2:**
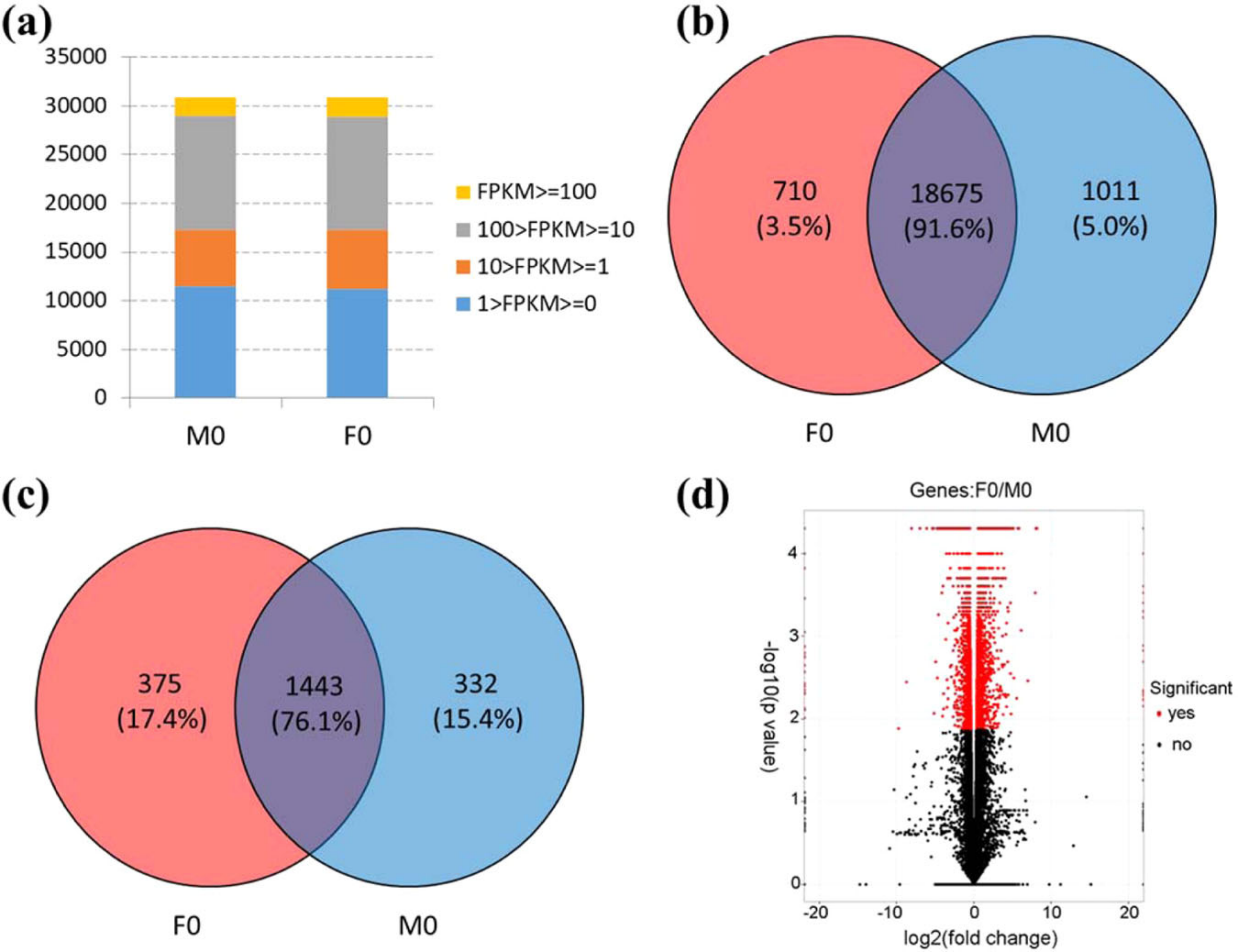
Overview of papaya M0 and F0 floral bud transcriptome. **a** Distribution of genes that displayed high expression (FPKM > = 100), middle expression (100 > FPKM > = 10), low expression (10 > FPKM > = 1), and no expression (1 > FPKM > = 0). **b** Venn diagram of genes expressed in male and female samples. **c** Venn diagram illustrating genes displayed high expression in at least one sample. **d** Volcano plot of transcriptomes of male and female floral buds. Red dots show the significant differential genes with FDR < 0.05

The cDNA libraries of M0 and F0 floral buds were constructed with three biological replications. In total, we obtained 222.9 million raw reads and 32.2–40.9 million reads per library (Table 1). We used Tophat and Cufflinks pipeline to align reads to the papaya reference genome and quantify expression levels of genes. Approximately 85% reads could be mapped to the reference genome, and about 78% paired reads were aligned concordantly. M0 and F0 shared similar distributions of number of genes in different expression level ranges (Fig. 2a). Nearly one third of the 30861 annotated genes displayed no expression in both samples, with FPKM values lower than one. Genes expressed at middle level (100 > FPKM > = 10) accounted for the highest proportion, followed by those expressed at low level (FPKM lower than 10), and the highly expressed genes (FPKM > 100) accounted for the least proportion. We further compared the expressed genes between M0 and F0, and found that M0 and F0 shared 91.6% of these genes and 1011 and 710 genes were specifically expressed in male and in female floral buds, respectively (Fig. 2b).

**Table 1:** Statistics of sequencing and mapping results of male and female floral bud libraries

Samples	Biological replicates	Raw reads	Overall read mapping rate	Aligned pairs	Concordant pair alignment rate	Multiple alignments	Multiple alignments rate	Discordant alignments	Discordant alignments rate
Male (M0)	1	38,063,298	85.50%	15065488	78.20%	1338788	8.90%	177971	1.20%
	2	39,460,518	85.40%	15598027	78.50%	485029	3.10%	116783	0.70%
	3	40,993,746	85.10%	16093470	77.90%	405777	2.50%	127459	0.80%
Female (F0)	1	39,686,726	84.30%	15509921	77.00%	2424819	14.90%	231476	1.50%
	2	32,243,508	83.40%	12286182	75.50%	514655	4.20%	121576	1.00%
	3	32,447,020	85.30%	12844031	77.70%	2098871	16.30%	235412	1.80%

### Annotation of highly expressed genes revealed rapid growth in male and female buds

A total of 1775 and 1818 highly expressed genes were detected in male and female floral buds, respectively, and 76.1% of them were shared between male and female floral buds (Fig. 2c). In order to reveal the functions of the highly expressed genes in female and male floral buds, we performed genome ontology (GO) enrichment analysis. We found that genes involved in cell proliferation such as nucleosome assembly, ribosome biogenesis, and protein metabolic processes, were enriched in both samples (Supplementary file 1: Fig. S1). This result suggested that floral buds at this developmental stage were undergoing rapid cell division and expansion. The Kyoto encyclopedia of genes and genomes (KEGG) pathway enrichment confirmed the above result, with ribosome and spliceosome overrepresented in both samples (Supplementary file 2: Table S1).

We also conducted GO term and KEGG analysis for the highly expressed genes showing sex-specific expression. RNA transport pathway was significantly enriched in female-specifically expressed genes, while proteasome pathway enriched in male-specifically expressed genes. In consistence with this, the GO category of modification-dependent protein catabolic process (proteolysis involved) was overrepresented in male-specifically expressed genes. In addition, the GO terms of water transport and proton transport were also enriched in male-specifically expressed genes, while 1,3-bata-glucan biosynthetic process was enriched in female-specifically expressed genes.

### Genes related to plant hormone signal transduction are significantly overrepresented in the DEGs between male and female floral buds

A total of 1734 DEGs were identified between M0 and F0, including 923 female-biased and 811 male-biased genes. To reveal the major function of these DEGs, GO annotation was performed. Among the 1734 DEGs, 1103 genes were annotated with at least one GO term and were categorized into the three main GO ontologies. For the molecular function, genes involved in the catalytic activity and binding were overrepresented. Within the biological process, genes involved in metabolic process, cellular process, and localization were enriched (Supplementary file 3: Table S2). We also used AgriGO to elucidate the functional enrichment of the DEGs, using a cutoff of FRD < = 0.05. Genes in four GO terms in cellar functions categories were significantly enriched and they were GO:0030312, external encapsulating structure; GO:0005618, cell wall; GO:0005576, extracellular region; GO:0045259, proton-transporting ATP synthase complex (Supplementary file 4: Table S3). KEGG pathway enrichment analysis showed that plant hormone signal transduction (ko04075) was significantly overrepresented in the DEGs. Among the 37 genes involved in this category, 16 are associated with auxin biosynthesis and signal transduction, 3 are associated with abscisic acid (ABA) signaling, and the remaining were related to other hormones, such as cytokinin and GA (Supplementary file 5: Table S4).

### Sex-biased genes are involved in hormone biosynthesis and signaling

To investigate the hormone function during the process of sexual dimorphism, hormone-related genes were identified in papaya. Forty-eight hormone-related genes exhibited differential expression between male and female floral buds (Table 2). Among them, 12 genes are involved in ABA biosynthesis and transportation. Ten of them showed female-biased expression, including the ABA biosynthesis enzyme ABA1 and the vital ABA acceptor PYR1. The other two genes showed male-biased expression and are involved in ABA signal transduction. Nine genes involved in auxin biosynthesis and signal transduction displayed sex-biased expression. Including the genes in auxin metabolism pathway annotated by KEGG, a total of 18 genes involved in auxin biosynthesis and signaling showed sex-biased expression.

**Table 2:** List of 48 differentially expressed hormone-related genes between male (M0) and female (F0) floral buds

Gene ID	Best hit in Arabidopsis	Hormone	Involved process	Expression level (FPKM)			*p*-value
				M0	F0	log2 (fold change)	
evm.TU.supercontig 55.145	ABA DEFICIENT 1 (ABA1)	Abscisic acid	Hormone biosynthesis	4.462	9.404	1.076	0.00085
evm.TU.supercontig 10.232	Related to AP2 6 (RAP2.6)	Abscisic acid	Hormone signal transduction	6.990	42.015	2.587	5.00E-05
evm.TU.supercontig 1195.3	WRKY DNA binding protein 2 (MYB2)	Abscisic acid	Hormone signal transduction	4.013	1.467	−1.452	0.0014
evm.TU.supercontig 1780.1	Heat shock transcription factor C1 (HSFC1)	Abscisic acid	Hormone signal transduction	5.172	14.244	1.461	5.00E-05
evm.TU.supercontig 92.51	GENOMES UNCOUPLED 5 (GUN5)	Abscisic acid	Hormone receptor	10.003	24.359	1.284	0.0002
evm.TU.supercontig 87.35	Multidrug resistance-associated protein 5 (MRP5)	Abscisic acid	Hormone signal transduction	11.057	26.980	1.287	0.0016
evm.TU.supercontig 6.420	Myo-inositol polyphosphate 5-phosphatase 2 (5PTASE2)	Abscisic acid	Hormone signal transduction	0.698	9.583	3.780	0.0052
evm.TU.contig 36665.1	PYRABACTIN RESISTANCE 1 (PYR1)	Abscisic acid	Hormone receptor	5.179	10.947	1.080	0.00385
evm.TU.supercontig 38.71	Related to AP2 6 (RAP2.6)	Abscisic acid	Hormone signal transduction	4.308	16.998	1.980	0.0001
evm.TU.supercontig 65.79	RING-H2 finger A2A (RHA2A)	Abscisic acid	Hormone signal transduction	40.908	18.099	−1.176	5.00E-05
evm.TU.supercontig 18.89	Long chain acyl CoA synthetase 2	Abscisic acid	Hormone signal transduction	286.530	135.281	1.083	5.00E-05
evm.TU.contig 36665.1	Polyketide cyclase/dehydrase and lipid transport superfamily protein	Abscisic acid	Hormone receptor	5.179	10.947	1.080	0.00385
evm.TU.supercontig 58.29	AUXIN RESISTANT 3 (AXR3)	Auxin	Hormone signal transduction	2.243	0.777	−1.529	0.0043
evm.TU.supercontig 292.1	Auxin-responsive GH3 family protein, BRU6	Auxin	Hormone metabolism	5.451	14.983	1.459	5.00E-05
evm.TU.supercontig 58.26	SHORT HYPOCOTYL 2 (SHY2)	Auxin	Hormone signal transduction	42.949	13.331	−1.688	5.00E-05
evm.TU.supercontig 26.242	Cytochrome P450, family 83, subfamily B, polypeptide 1, (CYP83B1)	Auxin	Hormone biosynthesis	15.449	0.550	−4.813	5.00E-05
evm.TU.contig 32826.1	Auxin-responsive GH3 family protein, WES1	Auxin	Hormone metabolism	12.166	45.988	1.918	5.00E-05
evm.TU.supercontig 78.79	Flavin-binding monooxygenase family protein YUCCA6 (YUC6)	Auxin	Hormone biosynthesis	0.844	1.876	1.151	0.00315
evm.TU.supercontig 36.134	Cryptochrome 1 (CRY1)	Auxin	Hormone signal transduction	11.856	36.953	1.640	5.00E-05
evm.TU.supercontig 1346.4	AUX/IAA transcriptional regulator family protein	Auxin	Hormone signal transduction	6.287	2.162	1.540	0.00025
evm.TU.supercontig 131.87	Tyrosine transaminase family protein (TAA)	Auxin	Hormone biosynthesis	2.837	15.224	2.424	0.0048
evm.TU.supercontig 14.94	Expansin A5 (EXPA5)	Brassinosteroid	Hormone signal transduction	71.440	27.609	−1.372	0.00015
evm.TU.supercontig 36.153	PHOSPHATE-INDUCED 1 (PHI-1)	Brassinosteroid	Hormone signal transduction	59.677	146.013	1.291	5.00E-05
evm.TU.supercontig 131.69	TCP family transcription factor	Brassinosteroid	Hormone signal transduction	7.526	25.648	1.769	5.00E-05
evm.TU.supercontig 13.46	TCP family transcription factor, TCP1	Brassinosteroid	Hormone signal transduction	1.319	13.040	3.306	0.0001
evm.TU.contig 33460.1	Sugar transporter 2	Brassinosteroid	Hormone signal transduction	2.016	0.166	3.603	0.0011
evm.TU.supercontig 209.43	Phosphate responsive 1 family protein	Brassinosteroid	Hormone signal transduction	358.522	818.756	1.191	5.00E-05
evm.TU.supercontig 182.24	Cytokinin oxidase/dehydrogenase 1 (CKX1)	Cytokinin	Hormone metabolism	1.682	3.938	1.227	0.00055
evm.TU.supercontig 5.323	Response regulator 4 (ARR4)	Cytokinin	Hormone signal transduction	6.788	14.432	1.088	5.00E-05
evm.TU.supercontig 16.85	Histidine containing phosphotransmitter 2	Cytokinin	Hormone signal transduction	71.867	159.385	1.149	0.00275
evm.TU.supercontig 80.38	Aluminium induced protein with YGL and LRDR motifs	Ethylene	Hormone response	150.469	437.319	1.539	5.00E-05
evm.TU.supercontig 750.1	EIN3-binding F box protein 1 (EBF1)	Ethylene	Hormone signal transduction	36.410	139.133	1.934	5.00E-05
evm.TU.supercontig 50.117	Glutathione S-transferase PHI 9 (GSTF9)	Ethylene	Hormone response	151.469	66.856	−1.180	0.00055
evm.TU.supercontig 23.98	Multiprotein bridging factor 1 C (MBF1C)	Ethylene	Hormone response	35.789	187.079	2.386	5.00E-05
evm.TU.supercontig 44.56	myb domain protein 72 (MYB72)	Ethylene	Hormone signal transduction	7.123	0.868	−3.036	0.00015
evm.TU.supercontig 49.68	Alcohol dehydrogenase 1 (ADH1)	Ethylene	Hormone response	7.053	37.928	2.427	5.00E-05
evm.TU.supercontig 20.69	Integrase type DNA binding superfamily protein	Ethylene	Hormone response	6.762	3.032	1.157	0.00515
evm.TU.supercontig 46.8	EIN3 binding F box protein 1	Ethylene	Hormone signal transduction	14.639	33.836	1.209	5.00E-05
evm.TU.supercontig 731.1	GA INSENSITIVE DWARF1A (GID1A)	Gibberellin	Hormone receptor	131.621	280.810	1.093	5.00E-05
evm.TU.supercontig 21.138	F-box family protein SLEEPY1 (SLY1)	Gibberellin	Hormone signal transduction	61.592	143.984	1.225	5.00E-05
evm.TU.supercontig 1055.1	Homeodomain-like superfamily protein LHY	Gibberellin	Hormone signal transduction	52.521	462.398	3.138	0.0033
evm.TU.supercontig 4.180	Gibberellin 20-oxidase 4 gibberellin 20-oxidase 4 (GA20OX4)	Gibberellin	Hormone biosynthesis	3.643	0.393	−3.214	0.0001
evm.TU.supercontig 84.117	Alpha/beta Hydrolases superfamily protein	Gibberellin	Hormone receptor	139.816	283.175	1.018	5.00E-05
evm.TU.contig 30289.2	myb domain protein 108 putative transcription factor MYB108	Jasmonic acid	Hormone signal transduction	2.146	8.424	1.973	5.00E-05
evm.TU.supercontig 49.67	Plant stearoyl-acyl-carrier-protein desaturase family protein SSI2	Jasmonic acid	Hormone biosynthesis	19.229	39.811	1.050	5.00E-05
evm.TU.supercontig 9.199	Auxin-responsive GH3 family protein AVRPPHB SUSCEPTIBLE 3 (PBS3)	Salicylic acid	Hormone signal transduction	22.894	10.712	−1.096	5.00E-05
evm.TU.supercontig 18.78	WRKY DNA-binding protein 8 member of WRKY Transcription Factor	Salicylic acid	Hormone signal transduction	3.085	10.767	1.803	5.00E-05
evm.TU.supercontig 23.45	Glutamine dumper 3	Salicylic acid	Hormone signal transduction	16.059	2.687	2.579	5.00E-05

The auxin biosynthesis gene *YUC6* and *tyrosine transaminase family protein* (*TAA*) in the IPyA synthesis pathway were expressed at a higher level in females than in males, while *cytochrome P450 family 83 subfamily B polypeptide 1* (*CYP83B1*) involved in the indole-3-acetaldoxime (IAOx) biosynthesis pathway showed an opposite expression pattern (Supplementary file 6: Fig. S2). *Cryptochrome-1* (*CRY 1*), an important gene involved in the polar transport of auxin, was expressed at a much higher level in females than in males. *GH3* family genes are auxin-responsive genes and participate in auxin homeostasis. Four *GH3* family genes were expressed at a much higher level in females than in males. The early auxin-responsive genes such as *AUX/IAA, ARP*, and *SAUR* also showed female-biased expression. *SHY2* and *AUXIN RESISTANT 3*, the *AUX/IAA* transcriptional regulators, showed male-biased expression (Supplementary file 6: Fig. S2). Auxin promotes the degradation of auxin response gene *Aux/IAAs*, and therefore a higher concentration of auxin would be expected in female than in male buds. In short, the key genes in the auxin biosynthesis and signaling pathways were differentially expressed between males and females.

Although ethylene had been demonstrated playing the key role in sex determination of melon, only 8 ethylene reception and transduction genes showed differential expression between papaya males and females. GA is a well-known masculine hormone. We identified five sex-biased genes that are involved in GA biosynthesis and signaling. All the five genes except one (low-expression in both females and males) showed female-biased expression. Moreover, nearly all the genes involved in BR, JA, and cytokinin biosynthesis and signaling showed a higher expression in females than in males (Table 2).

### Differentially expressed transcription factors between male and female buds

Transcription factors (TFs) play vital roles in flower development. Fifty-three TF genes belonging to 26 TF families exhibited significantly differential expression between males and females (Supplementary file 7: Table S5). AP2/EREBP family and MADS-box family were the most representative ones. Three out of five genes in the AP2/EREBP family exhibited a higher level of expression in females than in males. Three MADS-box genes showed male-biased expression, and one showed female-biased expression. Two genes from the MYB family displayed male-biased expression while three genes belonging to the MYB-related family displayed female-biased expression. Some gene families, such as HSF family and AUX/IAA family, displayed coordinated gene expression patterns.

### Transcription factor binding sites of hormone-related genes

We searched the transcription factor binding sites in the promoters of hormone-related DEGs. Six TF binding consensus sequences, MYB2, HSF1, Alfin1, GAMYB, DREB, and AHL12-3ary, were enriched in the hormone-related DEGs.

Most flower organ identity genes contain the MADS-box domain, which specifically recognizes the CArG-box DNA binding motifs. Among the 44 hormone-related genes, 20 of them, including biosynthesis genes of ABA (*zeaxanthin epoxidase*, *ZEP*), GA (*gibberellin 20-oxidase 4*, *GA20OX4*), and Jasmonates (*Plant stearoyl-acyl-carrier-protein desaturase family protein*, *SSI2*) contained at least one CArG-box binding site (Supplementary file 8: Table S6). Two ABA signal transduction genes, *MYB2* and *RAP2.6*, and several auxin-signal-related genes, including the important polar transport inhibitor *CRY1*, also contained CArG-box DNA binding motifs in their promoters. In addition, *ARF1* and *WRKY2* binding motifs were also identified in a large proportion of these genes.

**Fig. 3:**
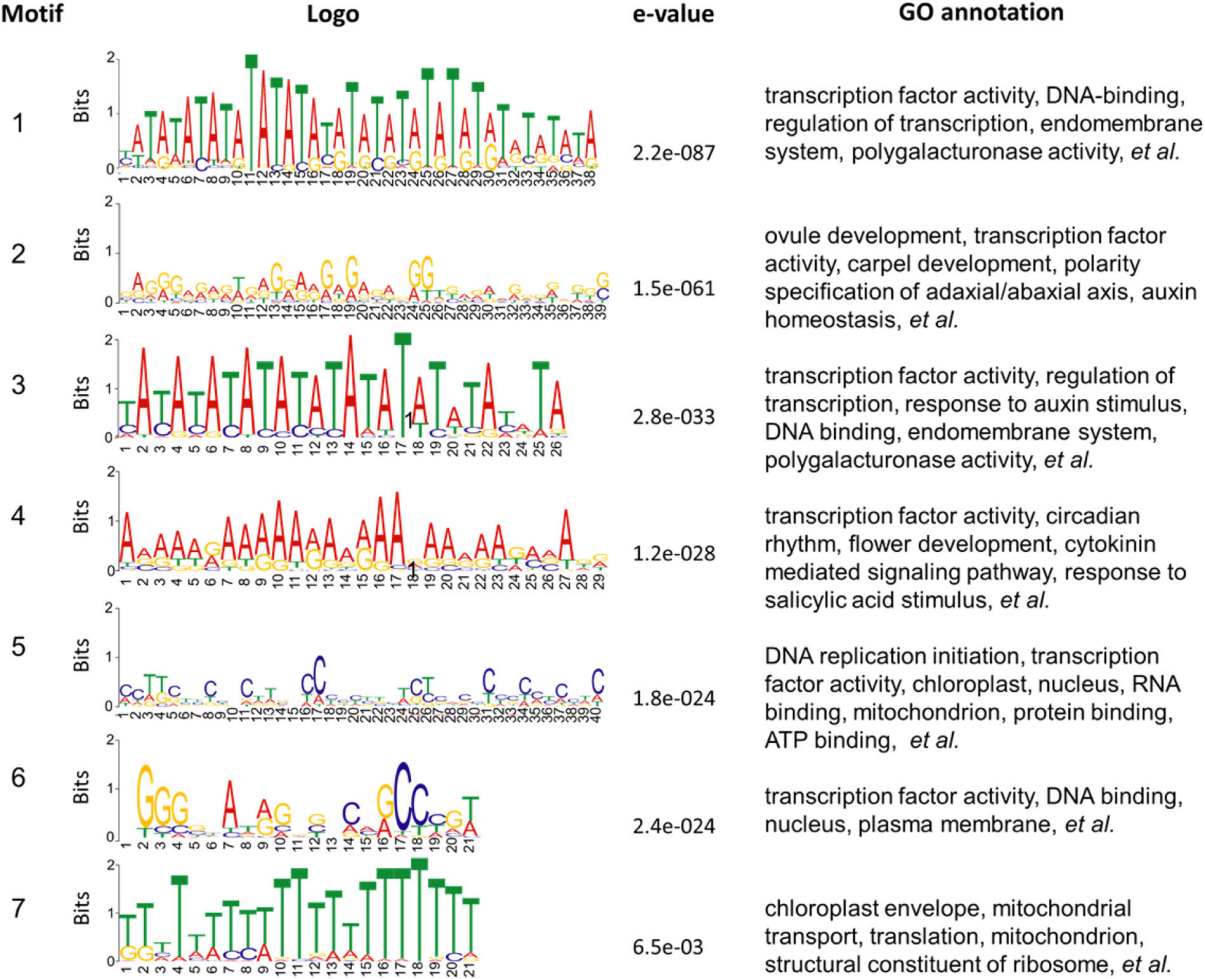
Motifs enriched in the promoter of hormone-related DEGs. De novo transcription factor binding motifs enriched in the 2k promoter of hormone-associated DEGs were detected by PROMO. The putative function were predicted by GOMO programs

Besides the known motifs, we also identified seven de novo motifs that are abundant in the promoter of these genes (Fig. 3). The potential roles of those motifs were predicted by GOMO using *Arabidopsis thaliana* (Plant) database. Six out of the seven motifs were significantly enriched in genes involved in TF activity and regulation of transcription. We then investigated the specific GO terms associated with each motif. Motif 1 and motif 3 are potentially involved in polygalacturonase activity. Motif 2 is probably involved in the pathways related to ovule development, auxin homeostasis, and abaxial cell fate specification. Some motifs might function in the hormone pathway, such as motif 3 might respond on auxin stimulus and motif 4 might respond to salicylic acid stimulus and function in cytokinin-mediated signaling pathway (Supplementary file 9: Table S7).

### The expression patterns of stamen and carpel developmental genes in the two sex-types

We used the genes that have been annotated in *Arabidopsis* to be involved in stamen and carpel development as a query to obtain their homologs in papaya by BLASTP. Among the 141 gene identified, ten genes were differentially expressed between male and female papayas. These ten genes include eight female-biased genes and two male-biased genes (Table 3). The expression of *CpCRC* was 15 times higher in females than in males. *CpCRC* showed increased expression during the carpel development, but nearly no expression during the stamen development (unpublished data).

**Table 3:** List of MADS-box genes and reproductive organ developmental genes in papaya

Gene	Name	FPKM in M0	FPKM in F0	log2 (fold_change)	Sex-biased	Function
evm.TU.supercontig_26.318	*CpPI*	137.46	153.83	−0.162	No	B-class MADS-box gene
evm.TU.supercontig_471.4	*CpSTK*	0.082	2.262	−4.794	Female-biased	C-class MADS-box gene
evm.TU.supercontig_50.72	*CpPLE*	28.698	33.95	−0.242	No	C-class MADS-box gene
evm.TU.supercontig_6.195	*CpTM6-2*	17.417	22.114	−0.344	No	B-class MADS-box gene
evm.TU.supercontig_6.198	*CpTM6-1*	88.62	69.634	0.348	No	B-class MADS-box gene
evm.TU.supercontig_63.22	*CpFUL1*	8.587	12.038	−0.487	No	A-class MADS-box gene
evm.TU.supercontig_43.69	*CpSEP3*	41.211	75.741	−0.878	Female-biased	E-class MADS-box gene
CpXYh2_X	*CpSERK2*	17.543	29.882	−0.768	Female-biased	Petal differentiation and expansion stage
evm.TU.contig_30608.1	*CpCRC*	6.822	92.949	−3.768	Female-biased	Petal differentiation and expansion stage
evm.TU.supercontig_2.284	*CpMEE33*	160.223	102.736	0.641	Male-biased	Petal differentiation and expansion stage
evm.TU.supercontig_26.91	*CpSEU*	115.422	157.675	−0.45	Female-biased	Petal differentiation and expansion stage
evm.TU.supercontig_350.1	*CpJAG*	2.056	9.678	−2.235	Female-biased	Stamen and carpel development stage
evm.TU.supercontig_59.41	*CpBAM1*	13.168	50.157	−1.929	Female-biased	Petal differentiation and expansion stage
evm.TU.supercontig_78.79	*CpYUC6*	0.844	1.876	−1.151	Female-biased	Petal differentiation and expansion stage
evm.TU.supercontig_97.45	*CpGPT1*	60.586	33.274	0.865	Male-biased	L mature pollen stage

Among the reported MADS-box genes in papaya^31,32^, the C class gene *SEEDSTICK (STK)* showed female-biased expression. The three B-class genes were highly expressed in both males and females, except *CpTM6-2*. The E class genes, *CpMADS1* and *CpMADS3*, which are phylogenetically close to *Arabidopsis AGL2* (*SEP1*) and *AGL4* (*SEP2*), showed similar levels of expression between males and females *SEP3* homolog (evm.TU.supercontig_43.69), the most well-known E-class gene, showed female-biased expression.

### DEGs in the non-recombining region contribute to sex differentiation

**Fig. 4:**
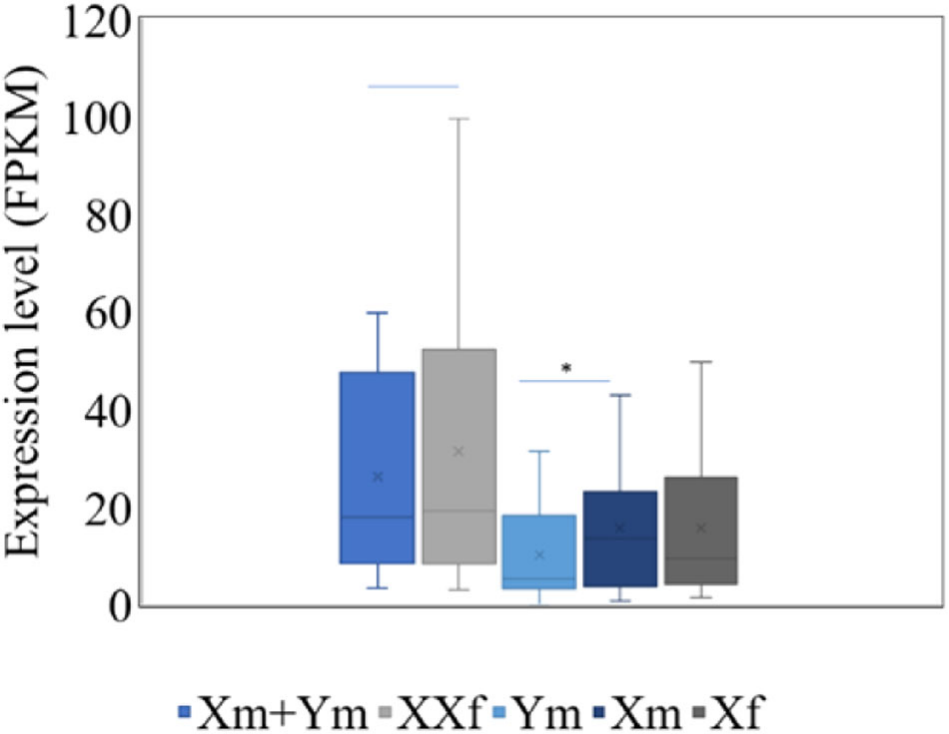
The expression level (FPKM) of alleles of pair genes in male and female. Xm X allele in male, Ym MSY allele in male, XXf two X allele in female, Xf one X allele in female

Among the 124 annotated genes on HSY and corresponding X regions, 81 did not express at stage 0 floral buds. Fifty-eight transcripts corresponding to 43 genes expressed (FPKM ≥ 1) at least in one sample. As expected, MSY alleles only expressed in male, whereas X alleles expressed in both sex types. The total expression of X and Y alleles of paired genes in males showed no significant difference with that of the two X alleles in females (Fig. 4). However, the expression of Y alleles was significantly lower than X alleles in males and lower than half expression of X alleles in females as well (Fig. 4). The two expressed X-specific genes also showed two times of expression in females relative to males. Seven Y-specific genes expressed specifically in males, including five coding genes and two pseudogenes, *PCpY-1* and *PCpY-9* (Supplementary file 10: Table S8).

In sex determinate regions, 11 genes showed significant differential expression between males and females, including two female-biased TFs, *CpXY2* and *CpXY9*. *CpXY2*, the ortholog of *AtSERK2*, is a member of receptor-like protein kinase (RLKs) gene family. *CpXY9*, the ortholog of *AtGeBP*, encodes a nuclear protein with DNA-binding activity. *CpXYh8_X* and *CpXYh11_X* are involved in methylation and regulation of chromosome structure and encode Sister chromatid cohesion 1 protein 4 and PWWP domain-containing protein, respectively.

### Validation of DEGs between male and female papaya floral buds

**Fig. 5:**
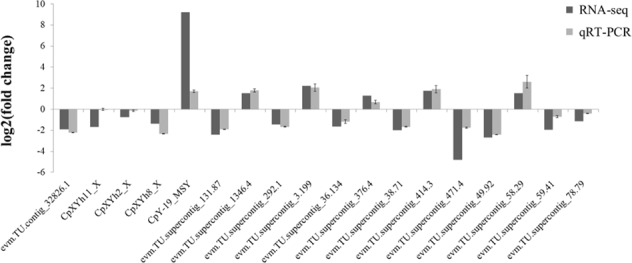
Validation of DEGs by qRT-PCR. Compare the expression level of DEGs between RNA-Seq and qRT-PCR

To confirm the differentially expressed genes, 18 DEGs were randomly selected for validation using quantitative real-time PCR (qPCR). Ubiquitin (UBQ) gene was used as a reference. Primers used for qPCR were list in Supplementary file 11: Table S9. We compared the results of qPCR with those of RNAseq and the expression patterns of these 18 genes were highly consistent between the two methods (Fig. 5).

## Discussion

Based on the widely accepted “two-gene” model, dioecy in flowering plants has evolved independently through at least two mutations, a male-sterility mutation (creating females) and a female-sterility mutation (creating males)^33^. Papaya female flowers belong to the type II unisexual flowers, which are unisexual from inception, whereas papaya male flowers belong to the type I unisexual flowers, which become unisexual by abortion of developing pistil. The type II flowers are likely resulted from mutations in a small subset of genes involved in the short pathway from floral commitment to reproductive organ identity, while the type I flowers are resulted from mutations in a large subset of genes involved in a wide range of developmental process of reproductive organ initiation and development^2^.

Comparative analysis of transcriptome profiles between male and female plants can effectively narrow down candidate genes for sex differentiation. However, the tissue types and developing stages selected for this type of study are very critical. We carefully examined the developmental stages of floral buds and dissected the flower buds at the stage of sepal and petal primordium for transcriptome analysis (Fig. 1). At this stage, there is no morphological difference between males and females, which eliminates the interference of sex-differentiation unrelated traits.

Flower organ identity genes, especially B and C class genes can be candidate genes responsible for sex differentiation in the type II unisexual flowers^2^. The B class genes are involved in sexual differentiation in *S. oleracea*^9,34^. In *S. oleracea*, the B class genes displayed male-specific expression pattern^9^ and silencing the B class genes resulted in homeotic transformation of stamens into carpels^34^. Similarly, knocking-down the B class gene, *ThdPI*, in *Thalictrum dioicum* resulted in male to female flower conversion^8^. Papaya female flower belongs to the type II unisexual flower and C class genes play essential roles in carpel identify. The C class gene *CpSTK* showed female sex-specific expression and likely plays important role in sex differentiation in papaya (Table 3). In contrast, the B class genes exhibited no expression difference between male and female at the early stage of flower development, suggesting that the B class genes do not play a major role in sex differentiation in papaya (Table 3).

Other genes regulating stamen or carpel development might also be involved in the sex differentiation in papaya. *AP2/EREBP* and MADS-box genes play vital roles in regulating flowering time and floral organ identity. Genes in these families were enriched among the differentially expressed TFs between males and females. Previous studies revealed that *CRABS CLAW* (*CRC*) genes are involved in the specification of carpel polarity and probably stigma and style formation^35,36^. *CpCRC* gene expression was more than 10 times higher in females than in males, suggesting its role in carpel morphogenesis (Table 3).

As principle signaling molecules, plant hormones play vital roles in regulating floral development and sex differentiation. Effects of plant hormones on sex differentiation vary between plant species. *CmASC7* and *CmACS11*, the sex determination genes in melon, encode the rate-limiting enzymes in ethylene biosynthesis^15–17^. In maize, brassinosteroid and jasmonate coordinately suppress the tassel development, while GA suppresses the stamen development^37,38^. In papaya, exogenous application of ethrel and auxin inhibitor can induce male-to-female sex reversal in papaya^39^.

It has been demonstrated that plant hormones are involved in sex regulation directly or indirectly through interaction and crosstalk with other hormones. GA promotes masculinization, while IAA, ethylene, and kinetin have feminization effect on sex differentiation^40,41^. ABA acts as an antagonist of GA in regulating developmental processes and environmental responses. It has been suggested that ABA participates in sex regulation by inhibiting GA activity^40,41^. We found that genes involved in the ABA biosynthesis and signaling transduction were significantly enriched in DEGs, and most of them showed a higher level of expression in females than in male plants (Table 2). Among them, Zeaxanthin epoxidase gene (*ABA1*) functions in the first step of the ABA biosynthesis, and *PYR1* acts as an ABA receptor. GA20OX4, a GA biosynthesis enzyme, displayed a higher level of expression in males than in females, suggesting its masculinization role in papaya. However, exogenous applications of GA_3_ on female and hermaphrodite papaya trees did not induce sex reversal phenotype but caused elongated peduncle^42^. The different levels of GA between male and female flowers might be the consequence rather than a cause of sex differentiation.

Auxin has been demonstrated to be involved in the initiation and subsequent development of floral organs^43^. Among the four interconnected Trp-dependent IAA biosynthesis pathways, synthesis via YUCCA (YUC) family of flavin monooxygenases was the predominant pathway for floral organ initiation and development^44,45^. In the IPyA pathway, indole-3-pyruvic acid (IPyA) is produced by TAA1 from Trp, and then converted into IAA by YUC. In *Arabidopsis*, high auxin concentration was detected in emerging stamen and carpel primordia^46^. *yuc1yuc4* double mutant displayed severe defects in all four whorls of floral organs, and no functional stamen or carpel organs were observed^45^. Rate-limiting enzymes of IAA biosynthesis, *TAA* and *YUC* showed a higher level of expression in females than in males, which may indicate active biosynthesis of auxin in the early stage of female floral development, even before the emergence of carpel primordia (Supplementary file 6: Fig. S2). In addition to local synthesis, auxin can be redistributed from the site of production to the site of action by transporters. *AtCRY1* has been reported as a vital suppressor of polar transport of auxin^47^. Auxin-responsive *GH3* family genes encode IAA-amino synthases that conjugates Asp and other amino acids to auxin in vitro^48^. The transcripts of these genes were relatively higher in female, also suggesting a higher level of auxin in female floral buds than in male floral buds (Supplementary file 6: Fig. S2). In addition, exogenous application of chlorflurenol, an inhibitor of auxin transport, could induce papaya male plants to produce female flowers^39^. All these suggested that auxin transporters might play a role in papaya sex differentiation.

Plant hormones coordinate plant growth and development through mutual interactions or crosstalk. Sex differentiation in papaya is likely under such coordinated regulation. We found that DNA binding sites of the GA-regulated transcription factor *GAMYB* were enriched in the promoters of hormone-related DEGs. This may suggest that crosstalk among hormones and signaling networks guides sex differentiation in papaya.

Epigenetic regulation by small RNA or DNA methylation could also play an important role in determining sex expression^10^. A Y-chromosome-encoded small RNA determined males in persimmons^49^. In *S. latifolia*, female sex suppression is modulated by methylation of specific DNA sequences^50^. We identified two DEGs in the sex determination region that are involved in methylation or chromatin structure modification, *CpXYh8_X* and *CpXYh11_X* (Supplementary file 10: Table S8). *CpXYh8_X* encodes sister chromatid cohesion 1 protein 4 and its homolog in *Arabidopsis*, *AtRAD21.3*, functions in sister chromatid cohesion during meiosis and DNA repair^51^. *CpXYh11_X* encodes a PWWP domain-containing protein, which are involved in chromatin-associated biological processes, such as transcriptional regulation and DNA repair^52^. The PWWP domain is also presented in mammalian DNA methyltransferases, such as *Dnmt3b* and *Dnmt3a* and these PWWP domain-containing proteins are involved in DNA methylation of the major satellite repeats at pericentric heterochromatin^53,54^.

## Supplementary information

Supplemental file 1

Supplemental file 2

Supplemental file 3

Supplemental file 4

Supplemental file 5

Supplemental file 7

Supplemental file 8

Supplemental file 9

Supplemental file 10

Supplemental file 11

Supplemental file 6
