# Supplementary material for: Sex biased expression of hormone related genes at early stage of sex differentiation in papaya flowers
Source: Hortic Res. 2021 Jul 1;8:147. doi: 10.1038/s41438-021-00581-4 (PMC8245580; doi:10.1038/s41438-021-00581-4)
Supplement: Supplementary file 2 — Supplemental file 2 [file 41438_2021_581_MOESM2_ESM.pdf]

The KEGG term that overrepresented in the high expressed genes in male (M0) and female (F0) floral buds.

Sample	KEGG term	Database	ID	Input number	Background number	P-Value
F0	Ribosome	KEGG PATHWAY	ko03010	159	266	1.84E-32
	Spliceosome	KEGG PATHWAY	ko03040	65	167	6.56E-08
	RNA transport	KEGG PATHWAY	ko03013	48	172	0.00221629
	Photosynthesis	KEGG PATHWAY	ko00195	20	52	0.002585759
	Photosynthesis - antenna	KEGG PATHWAY	ko00196	9	16	0.006447443
	Phagosome	KEGG PATHWAY	ko04145	23	73	0.009505304
M0	Ribosome	KEGG PATHWAY	ko03010	161	266	1.94E-28
	Proteasome	KEGG PATHWAY	ko03050	25	46	5.76E-05
	Phagosome	KEGG PATHWAY	ko04145	29	73	0.00101881
	Photosynthesis - antenna	KEGG PATHWAY	ko00196	11	16	0.001822633
	Spliceosome	KEGG PATHWAY	ko03040	50	167	0.003970078
	Photosynthesis	KEGG PATHWAY	ko00195	20	52	0.007656939
