# Supplementary material for: Sex biased expression of hormone related genes at early stage of sex differentiation in papaya flowers
Source: Hortic Res. 2021 Jul 1;8:147. doi: 10.1038/s41438-021-00581-4 (PMC8245580; doi:10.1038/s41438-021-00581-4)
Supplement: Supplementary file 3 — Supplemental file 3 [file 41438_2021_581_MOESM3_ESM.pdf]

Level 2 Gene Ontology annotation of 1734 DEGs between male and female in ontologies.

Total gene number with GO ID: 1099

Term type	Function description	No.	Percentage
Cellular Component	extracellular region	17	1.5
	extracellular region part	3	0.3
	cell	215	19.5
	cell part	215	19.5
	membrane-enclosed lumen	2	0.2
	envelope	3	0.3
	macromolecular complex	30	2.7
	organelle	120	10.9
	organelle part	31	2.8
	symplast	2	0.2
Biological Process	cellular component biogenesis	6	0.5
	developmental process	12	1.1
	reproduction	8	0.7
	cellular component organization	7	0.6
	obsolete biological process	4	0.4
	reproductive process	6	0.5
	immune system process	1	0.1
	response to stimulus	54	4.9
	multicellular organismal process	17	1.5
	multiorganism process	3	0.3
	establishment of localization	72	6.5
	metabolic process	267	24.2
	pigmentation	25	2.3
	localization	73	6.6
	cellular process	237	21.5
	biological regulation	30	2.7
Molecular Function	electron carrier activity	1	0.1
	nutrient reservoir activity	2	0.2
	enzyme regulator activity	2	0.2
	catalytic activity	191	17.3
	structural molecule activity	2	0.2
	binding	211	19.1
	transporter activity	6	0.5

ı three main

GO
GO:0005576
GO:0044421
GO:0005623
GO:0044464
GO:0031974
GO:0031975
GO:0032991
GO:0043226
GO:0044422
GO:0055044
GO:0044085
GO:0032502
GO:0000003
GO:0016043
GO:0008371
GO:0022414
GO:0002376
GO:0050896
GO:0032501
GO:0051704
GO:0051234
GO:0008152
GO:0043473
GO:0051179
GO:0009987
GO:0065007
GO:0009055
GO:0045735
GO:0030234
GO:0003824
GO:0005198
GO:0005488
GO:0005215
