# Supplementary material for: Sex biased expression of hormone related genes at early stage of sex differentiation in papaya flowers
Source: Hortic Res. 2021 Jul 1;8:147. doi: 10.1038/s41438-021-00581-4 (PMC8245580; doi:10.1038/s41438-021-00581-4)
Supplement: Supplementary file 4 — Supplemental file 4 [file 41438_2021_581_MOESM4_ESM.pdf]

Significantly enriched GO terms of total DEGs between male and female floral buds analyzed by AgriGO.

GO term	Ontology	Description	Number in input list	Number in BG/Ref	p-value	FDR
GO:0030312	C	external encapsulating structure	33	170	4.20E-06	0.00072
GO:0005618	C	cell wall	33	168	3.20E-06	0.00072
GO:0005576	C	extracellular region	60	379	1.50E-06	0.00072
GO:0045259	C	proton-transporting ATP synthase complex	8	20	0.00011	0.015
