# Supplementary material for: Sex biased expression of hormone related genes at early stage of sex differentiation in papaya flowers
Source: Hortic Res. 2021 Jul 1;8:147. doi: 10.1038/s41438-021-00581-4 (PMC8245580; doi:10.1038/s41438-021-00581-4)
Supplement: Supplementary file 5 — Supplemental file 5 [file 41438_2021_581_MOESM5_ESM.pdf]

List of DEGs that enriched in plant hormones synthesis and signal transduction pathway

Query	Gene ID	Gene Name	Hormone
evm.TU.supercontig_81.92	K14497	PP2C	Abscisic acid
evm.TU.contig_36665.1	K14496	PYL	Abscisic acid
evm.TU.supercontig_273.7	K14496	PYL	Abscisic acid
evm.TU.supercontig_9.166	K14485	TIR1	Auxin
evm.TU.contig_35052.1	K14484	IAA	Auxin
evm.TU.supercontig_6.73	K14487	GH3	Auxin
evm.TU.supercontig_10.158	K14484	IAA	Auxin
evm.TU.supercontig_9.199	K14487	GH3	Auxin
evm.TU.supercontig_37.54	K14488	SAUR	Auxin
evm.TU.supercontig_26.30	K14488	SAUR	Auxin
evm.TU.supercontig_34.118	K14487	GH3	Auxin
evm.TU.contig_32826.1	K14487	GH3	Auxin
evm.TU.supercontig_58.26	K14484	IAA	Auxin
evm.TU.supercontig_1065.2	K14487	GH3	Auxin
evm.TU.supercontig_19.207	K13946	AUX1	Auxin
evm.TU.supercontig_261.2	K14486	ARP	Auxin
evm.TU.supercontig_58.29	K14484	IAA	Auxin
evm.TU.supercontig_1346.4	K14484	IAA	Auxin
evm.TU.supercontig_292.1	K14487	GH3	Auxin
evm.TU.supercontig_48.217	K14488	SAUR	Auxin
evm.TU.supercontig_233.22	K14504	TCH4	Brassinosteroid
evm.TU.supercontig_233.23	K14504	TCH4	Brassinosteroid
evm.TU.supercontig_49.5	K14499	BKI1	Brassinosteroid
evm.TU.supercontig_9.100	K14491	ARR-B	Cytokinin
evm.TU.supercontig_5.323	K14492	ARR-A	Cytokinin
evm.TU.supercontig_16.85	K14490	AHP	Cytokinin
evm.TU.supercontig_84.119	K14490	AHP	Cytokinin
evm.TU.supercontig_46.8	K14515	EBF1_2	Ethylene
evm.TU.supercontig_750.1	K14515	EBF1_2	Ethylene
evm.TU.supercontig_21.138	K14495	GID2	Gibberelin
evm.TU.supercontig_116.43	K14494	DELLA	Gibberelin
evm.TU.supercontig_731.1	K14493	GID1	Gibberelin
evm.TU.supercontig_84.117	K14493	GID1	Gibberelin
evm.TU.supercontig_116.63	K13464	JAZ	Jasmonic acid
evm.TU.supercontig_207.8	K13464	JAZ	Jasmonic acid
evm.TU.supercontig_20.82	K13449	PR1	Salicylic acid
evm.TU.supercontig_5.187	K14508	NPR1	Salicylic acid
