# Supplementary material for: Sex biased expression of hormone related genes at early stage of sex differentiation in papaya flowers
Source: Hortic Res. 2021 Jul 1;8:147. doi: 10.1038/s41438-021-00581-4 (PMC8245580; doi:10.1038/s41438-021-00581-4)
Supplement: Supplementary file 6 — Supplemental file 7 [file 41438_2021_581_MOESM6_ESM.pdf]

List of transcription factors that showed sex-biased expression between male and female floral buds.

Gene ID	M0 (FPKM)	F0 (FPKM)	log2(fold change)	E-value	Domain	Family	Annotation
evm.TU.supercontig_10.232	6.990	42.015	2.587	5.00E-05	AP2	AP2-EREBP	ethylene-responsive transcription factor win1-like
evm.TU.supercontig_20.69	6.762	3.032	-1.157	0.00515	AP2	AP2-EREBP	c-repeat dehydration-responsive element-binding factor 4
evm.TU.supercontig_38.71	4.308	16.998	1.980	0.0001	AP2	AP2-EREBP	ethylene-responsive transcription factor erf113-like
evm.TU.supercontig_49.92	3.349	21.708	2.696	5.00E-05	AP2	AP2-EREBP	ap2 domain-containing transcription factor family protein
evm.TU.supercontig_89.67	3.261	0.757	-2.107	0.00115	AP2	AP2-EREBP	integrase-type dna-binding superfamily
evm.TU.supercontig_1346.4	6.287	2.162	-1.540	0.00025	AUX_IAA	AUX/IAA	auxin-induced protein iaa4
evm.TU.supercontig_58.26	42.949	13.331	-1.688	5.00E-05	AUX_IAA	AUX/IAA	aux iaa transcriptional regulator family protein
evm.TU.supercontig_58.29	2.243	0.777	-1.529	0.0043	AUX_IAA	AUX/IAA	auxin-responsive protein iaa14-like
evm.TU.supercontig_21.8	5.076	11.253	1.149	5.00E-05	HLH	bHLH	transcription factor bhlh30-like
evm.TU.supercontig_84.89	2.420	12.414	2.359	0.0039	HLH	bHLH	dna binding isoform 2
evm.TU.supercontig_244.3	27.345	65.032	1.250	5.00E-05	bZIP_1	bZIP	basic leucine zipper 9
evm.TU.supercontig_9.71	2.914	13.658	2.229	5.00E-05	bZIP_1	bZIP	bzip family transcription factor family protein
evm.TU.supercontig_3.508	5.557	0.370	-3.908	5.00E-05	CCT	C2C2-CO-like	zinc finger protein constans-like 16
evm.TU.supercontig_70.6	21.438	161.081	2.910	5.00E-05	CCT	C2C2-CO-like	constans-like 1 protein
evm.TU.supercontig_111.11	3.920	14.603	1.897	5.00E-05	zf-Dof	C2C2-Dof	dof zinc finger protein
evm.TU.supercontig_1427.1	427.525	1263.650	1.564	5.00E-05	zf-CCCH	C3H	zinc finger ccch domain-containing protein 20-like
evm.TU.supercontig_136.30	6.926	15.282	1.142	5.00E-05	CBFD_NFYB_HMF	CCAAT	nuclear transcription factor y subunit b-3
evm.TU.supercontig_55.145	4.462	9.404	1.076	0.00085	FAD_binding_3	FHA	zeaxanthin epoxidase isoform 2
evm.TU.supercontig_10.71	3.033	9.087	1.583	5.00E-05	Acetyltransf_1	GNAT	acyl- n-acyltransferases superfamily protein
evm.TU.supercontig_9.59	1.354	0.313	-2.116	0.00295	Acetyltransf_1	GNAT	acyl- n-acyltransferases superfamily protein
evm.TU.supercontig_165.22	1.800	0.838	-1.104	0.00515	GRAS	GRAS	protein scarecrow-like
evm.TU.supercontig_8.249	0.262	1.495	2.514	0.00215	GRAS	GRAS	scarecrow-like protein 32
evm.TU.supercontig_107.24	0.427	3.451	3.014	0.0003	Homeobox	HB	homeobox-leucine zipper family protein lipid-binding start domain-containing isoform 2
evm.TU.supercontig_129.54	19.478	46.930	1.269	5.00E-05	HALZ	HB	homeodomain-leucine zipper protein hd4
evm.TU.supercontig_48.160	73.227	28.696	-1.352	5.00E-05	ELK	HB	homeobox protein knotted-1-like 1
evm.TU.supercontig_6.359	22.762	11.190	-1.024	5.00E-05	Homeobox	HB	homeobox-leucine zipper family protein
evm.TU.supercontig_69.79	156.814	71.824	-1.127	5.00E-05	KNOX1	HB	homeobox protein knotted-1-like 6
evm.TU.supercontig_1780.1	5.172	14.244	1.461	5.00E-05	HSF_DNA-bind	HSF	heat stress transcription factor C1
evm.TU.supercontig_55.149	35.792	82.621	1.207	5.00E-05	HSF_DNA-bind	HSF	heat shock factor protein hsf B1
evm.TU.supercontig_142.33	102.032	30.715	-1.732	5.00E-05	LIM	LIM	pollen-specific protein
evm.TU.supercontig_192.6	9.156	3.856	-1.248	0.001	DUF260	LOB	lob domain-containing 4 -like protein
evm.TU.supercontig_118.27	5.324	15.088	1.503	0.0001	SRF-TF	MADS	agamous-like mads-box protein agl80
evm.TU.supercontig_3.199	44.832	9.618	-2.221	5.00E-05	K-box	MADS	mads-box protein cmb1-like
evm.TU.supercontig_3486.1	1858.500	581.396	-1.677	5.00E-05	SRF-TF	MADS	agamous like6-like isoform partial
evm.TU.supercontig_414.3	30.118	8.815	-1.773	5.00E-05	K-box	MADS	agamous-like mads-box protein agl6
evm.TU.supercontig_23.98	35.789	187.079	2.386	5.00E-05	HTH_3	MBF1	multiprotein-bridging factor 1c
evm.TU.supercontig_112.57	5.429	0.636	-3.093	5.00E-05	Myb_DNA-binding	MYB	transcription factor divaricata
evm.TU.supercontig_376.4	37.785	15.400	-1.295	5.00E-05	Myb_DNA-binding	MYB	transcription factor myb44-like
evm.TU.supercontig_1055.1	52.521	462.398	3.138	0.0033	Myb_DNA-binding	MYB-related	homeodomain-like superfamily protein isoform 1
evm.TU.supercontig_178.23	2.499	31.377	3.650	5.00E-05	Myb_DNA-binding	MYB-related	protein reveille 1 isoform x2
evm.TU.supercontig_5.308	4.403	11.054	1.328	0.0047	Myb_DNA-binding	MYB-related	myb-like transcription factor family protein
evm.TU.supercontig_1.49	0.527	26.591	5.656	5.00E-05	NAM	NAC	nac domain ipr003441
evm.TU.supercontig_80.95	20.193	127.149	2.655	5.00E-05	NAM	NAC	nac domain class transcription factor isoform 1
evm.TU.supercontig_5.323	6.788	14.432	1.088	5.00E-05	Response_reg	Orphans	two-component response regulator
evm.TU.supercontig_6.215	33.037	146.164	2.145	0.00155	zf-B_box	Orphans	b-box zinc finger protein 19
evm.TU.supercontig_77.62	9.594	20.722	1.111	0.0012	zf-B_box	Orphans	light-regulated zinc finger protein isoform 1
evm.TU.supercontig_11.28	49.077	106.983	1.124	5.00E-05	Helicase_C	SNF2	atp-dependent helicase brm isoform x2
evm.TU.supercontig_34.18	23.803	49.151	1.046	5.00E-05	Helicase_C	SNF2	protein photoperiod-independent early flowering 1 isoform x1
evm.TU.supercontig_5.254	31.465	65.051	1.048	5.00E-05	TCP	TCP	transcription factor tcp11

evm.TU.supercontig_116.63	110.804	23.844	-2.216	5.00E-05	CCT_2	Tify	uncharacterized protein isoform 1
evm.TU.supercontig_130.33	22.943	46.618	1.023	5.00E-05	trihelix	Trihelix	trihelix transcription factor gt-1
evm.TU.supercontig_21.192	5.618	1.905	-1.561	5.00E-05	trihelix	Trihelix	trihelix transcription factor gt-2-like
evm.TU.supercontig_18.78	3.085	10.767	1.803	5.00E-05	WRKY	WRKY	probable wrky transcription factor 23
