# Supplementary material for: Sex biased expression of hormone related genes at early stage of sex differentiation in papaya flowers
Source: Hortic Res. 2021 Jul 1;8:147. doi: 10.1038/s41438-021-00581-4 (PMC8245580; doi:10.1038/s41438-021-00581-4)
Supplement: Supplementary file 7 — Supplemental file 8 [file 41438_2021_581_MOESM7_ESM.pdf]

List of 20 hormone-related genes that containing CArG motifs in their 2 kb promoter sequences.

Gene ID	Start position	End position	Dissimilarity	String	RE equally	RE query	Gene	Related-hormone
evm.TU.supercontig 10.232	701	710	12.836461	CCAAGTAGGG	0.12016	0.07921	AP2.6	ABA
evm.TU.supercontig 1195.3	688	697	4.628418	CCTTTTTTGG	0.02861	0.00724	MYB2	ABA
evm.TU.supercontig 55.145	612	621	4.628418	CCATTTTTGG	0.02861	0.00724	ZEP	ABA
evm.TU.contig 32826.1	1922	1931	4.628418	CCAAAGATGG	0.02861	0.00724	GH3	auxin
evm.TU.contig 32826.1	1982	1991	12.836461	CCTTATTTTG	0.12016	0.07921	GH3	auxin
evm.TU.supercontig 36.134	679	688	13.724397	CCGAATTTGG	0.04005	0.01352	CRY1	auxin
evm.TU.supercontig 36.153	1680	1689	13.724397	CCGTATTTTG	0.04005	0.01352	CRY1	auxin
evm.TU.supercontig 58.26	560	569	12.836461	GCTTATTTGG	0.12016	0.0698	SHY/IAA3	auxin
evm.TU.supercontig 58.26	560	569	12.836461	GCTTATTTGG	0.12016	0.07921	SHY/IAA3	auxin
evm.TU.supercontig_1065.2	1164	1173	10.144772	CCGTTTGTGG	0.00763	0.00052	GH3.17	auxin
evm.TU.supercontig_1065.2	332	341	13.724397	CAGTATTTGG	0.04005	0.01217	GH3.17	auxin
evm.TU.supercontig_26.30	667	676	12.836461	CCCCATTTGG	0.12016	0.0698	SAUR	auxin
evm.TU.supercontig_261.2	597	606	12.836461	CTAAATATGG	0.12016	0.0698	ARF	auxin
evm.TU.supercontig_261.2	1266	1275	4.628418	CCATACATGG	0.02861	0.00663	ARF	auxin
evm.TU.supercontig_37.54	661	670	12.836461	CTAAATAGGG	0.12016	0.0698	SAUR	auxin
evm.TU.supercontig_37.54	1937	1946	2.314209	CCAAACAAGG	0.01144	0.00349	SAUR	auxin
evm.TU.supercontig_48.217	1523	1532	13.724397	CCAAATACGT	0.04005	0.01217	SAUR	auxin
evm.TU.supercontig_48.217	1661	1670	6.942627	CCTTTTATGG	0.02289	0.00438	SAUR	auxin
evm.TU.supercontig_6.73	398	407	9.256836	CCCTTTCTGG	0.02289	0.00297	GH3.17	auxin
evm.TU.supercontig 131.69	568	577	4.628418	CCCTTTTTGG	0.02861	0.00724	TCP	BA
evm.TU.supercontig 131.69	719	728	4.628418	CCAAAAAAGG	0.02861	0.00724	TCP	BA
evm.TU.supercontig 131.69	1578	1587	4.628418	CCAAAGAGGG	0.02861	0.00724	TCP	BA
evm.TU.supercontig 14.94	1815	1824	12.836461	CCTTATTAGG	0.12016	0.07921	EXPA5	BA
evm.TU.supercontig 46.8	937	946	6.942627	CCATAAAGGG	0.02289	0.00479	EIN3	ethylene
evm.TU.supercontig 49.68	999	1008	9.256836	CCACAAATGG	0.02289	0.00318	ADH1	ethylene
evm.TU.supercontig 49.68	1482	1491	12.836461	TCATATTTGG	0.12016	0.07921	ADH1	ethylene
evm.TU.supercontig 4.180	640	649	12.836461	TCCTATTTGG	0.12016	0.07921	GA20OX4	GA
evm.TU.contig 30289.2	1321	1330	13.724397	ACGTATTTGG	0.04005	0.01352	MYB108	JA
evm.TU.contig 30289.2	1405	1414	12.836461	CCAAATAGGT	0.12016	0.07921	MYB108	JA
evm.TU.supercontig 49.67	42	51	12.836461	CTAAATATGG	0.12016	0.07921	SSI2	JA
evm.TU.supercontig 49.67	60	69	13.724397	CCAAATACAG	0.04005	0.01352	SSI2	JA
