# Supplementary material for: Sex biased expression of hormone related genes at early stage of sex differentiation in papaya flowers
Source: Hortic Res. 2021 Jul 1;8:147. doi: 10.1038/s41438-021-00581-4 (PMC8245580; doi:10.1038/s41438-021-00581-4)
Supplement: Supplementary file 8 — Supplemental file 9 [file 41438_2021_581_MOESM8_ESM.pdf]

The GO annotation of novel motifs enriched in the promoter of hormone-related genes. The novel motifs were identified by MEME program PROMO.

Motif	GO term	score	*p* -value	*q* -value	Specificity	GO Annotation	
1	GO:0003700	1.82E-07	2.65E-07	2.00E-04	~83%	MF	transcription factor activity
1	GO:0006355	4.55E-03	2.65E-07	2.00E-04	~8%	BP	regulation of transcription, DNA-dependent
1	GO:0045449	7.50E-04	2.65E-07	2.00E-04	~6%	BP	regulation of transcription
1	GO:0003677	7.42E-03	2.65E-07	2.00E-04	~3%	MF	DNA binding
1	GO:0012505	2.88E-05	2.65E-07	2.00E-04	~2%	CC	endomembrane system
1	GO:0004650	2.83E-02	4.22E-05	2.65E-02	100%	MF	polygalacturonase activity
2	GO:0006270	2.86E-02	5.09E-05	1.10E-02	~95%	BP	DNA replication initiation
2	GO:0035196	3.14E-02	7.53E-05	1.39E-02	~95%	BP	production of miRNAs involved in gene silencing by miRNA
2	GO:0048481	1.94E-02	1.11E-05	3.08E-03	~86%	BP	ovule development
2	GO:0003700	3.15E-08	2.65E-07	8.90E-05	~83%	MF	transcription factor activity
2	GO:0006355	7.72E-03	2.65E-07	8.90E-05	~8%	BP	regulation of transcription, DNA-dependent
2	GO:0004842	4.24E-02	2.79E-04	3.81E-02	~67%	MF	ubiquitin-protein ligase activity
2	GO:0009535	1.37E-02	1.59E-06	4.89E-04	~66%	CC	chloroplast thylakoid membrane
2	GO:0048440	3.34E-02	1.00E-04	1.68E-02	~61%	BP	carpel development
2	GO:0045449	1.59E-03	2.65E-07	8.90E-05	~6%	BP	regulation of transcription
2	GO:0010051	2.83E-02	4.93E-05	1.10E-02	~55%	BP	xylem and phloem pattern formation
2	GO:0003677	1.84E-06	2.65E-07	8.90E-05	~3%	MF	DNA binding
2	GO:0016563	1.96E-02	1.17E-05	3.08E-03	~29%	MF	transcription activator activity
2	GO:0005634	1.91E-05	2.65E-07	8.90E-05	~2%	CC	nucleus
2	GO:0003723	4.48E-02	3.45E-04	4.55E-02	~2%	MF	RNA binding
2	GO:0010154	3.34E-02	9.94E-05	1.68E-02	~19%	BP	fruit development
2	GO:0006468	2.41E-02	2.84E-05	6.98E-03	~18%	BP	protein amino acid phosphorylation
2	GO:0005739	3.72E-02	1.64E-04	2.42E-02	~12%	CC	mitochondrion
2	GO:0004674	2.90E-02	5.38E-05	1.10E-02	~11%	MF	protein serine/threonine kinase activity
2	GO:0005886	9.66E-03	2.65E-07	8.90E-05	~1%	CC	plasma membrane
2	GO:0005524	1.53E-03	2.65E-07	8.90E-05	100%	MF	ATP binding
2	GO:0009944	3.06E-02	6.58E-05	1.28E-02	100%	BP	polarity specification of adaxial/abaxial axis
2	GO:0010158	3.49E-02	1.22E-04	1.89E-02	100%	BP	abaxial cell fate specification
2	GO:0010599	3.50E-02	1.23E-04	1.89E-02	100%	BP	production of lsiRNA involved in RNA interference
2	GO:0010252	3.88E-02	1.97E-04	2.79E-02	100%	BP	auxin homeostasis
2	GO:0003777	1.02E-02	2.65E-07	8.90E-05	80%	MF	microtubule motor activity
2	GO:0048366	9.36E-03	2.65E-07	8.90E-05	40%	BP	leaf development

2	GO:0009507	1.56E-06	2.65E-07	8.90E-05	20%	CC	chloroplast
2	GO:0005515	7.33E-04	2.65E-07	8.90E-05	~0%	MF	protein binding
3	GO:0003700	4.07E-07	2.65E-07	2.50E-04	~83%	MF	transcription factor activity
3	GO:0006355	2.05E-02	8.75E-06	5.50E-03	~8%	BP	regulation of transcription, DNA-dependent
3	GO:0045449	3.12E-03	2.65E-07	2.50E-04	~6%	BP	regulation of transcription
3	GO:0009733	1.78E-02	3.71E-06	2.80E-03	~43%	BP	response to auxin stimulus
3	GO:0003677	8.76E-03	2.65E-07	2.50E-04	~3%	MF	DNA binding
3	GO:0012505	1.93E-06	2.65E-07	2.50E-04	~2%	CC	endomembrane system
3	GO:0004650	2.95E-02	5.12E-05	2.76E-02	100%	MF	polygalacturonase activity
4	GO:0003700	4.78E-09	2.65E-07	8.96E-05	~83%	MF	transcription factor activity
4	GO:0006355	1.28E-03	2.65E-07	8.96E-05	~8%	BP	regulation of transcription, DNA-dependent
4	GO:0004842	4.30E-02	2.46E-04	3.66E-02	~67%	MF	ubiquitin-protein ligase activity
4	GO:0045449	5.34E-04	2.65E-07	8.96E-05	~6%	BP	regulation of transcription
4	GO:0007623	3.64E-02	1.26E-04	2.24E-02	~6%	BP	circadian rhythm
4	GO:0004722	3.18E-02	7.16E-05	1.66E-02	~54%	MF	protein serine/threonine phosphatase activity
4	GO:0009965	4.09E-02	2.01E-04	3.11E-02	~52%	BP	leaf morphogenesis
4	GO:0004672	3.62E-02	1.21E-04	2.24E-02	~5%	MF	protein kinase activity
4	GO:0004702	4.57E-02	3.27E-04	4.68E-02	~34%	MF	receptor signaling protein serine/threonine kinase activity
4	GO:0003677	1.03E-04	2.65E-07	8.96E-05	~3%	MF	DNA binding
4	GO:0016563	1.65E-02	4.24E-06	1.21E-03	~29%	MF	transcription activator activity
4	GO:0005634	1.85E-05	2.65E-07	8.96E-05	~2%	CC	nucleus
4	GO:0007010	3.84E-02	1.52E-04	2.57E-02	~2%	BP	cytoskeleton organization
4	GO:0006468	5.53E-04	2.65E-07	8.96E-05	~18%	BP	protein amino acid phosphorylation
4	GO:0009908	2.53E-02	2.41E-05	5.98E-03	~13%	BP	flower development
4	GO:0007169	2.89E-04	2.65E-07	8.96E-05	~12%	BP	transmembrane receptor protein tyrosine kinase signaling pathway
4	GO:0043565	1.36E-02	1.59E-06	4.93E-04	~12%	MF	sequence-specific DNA binding
4	GO:0004674	1.91E-04	2.65E-07	8.96E-05	~11%	MF	protein serine/threonine kinase activity
4	GO:0005886	3.94E-05	2.65E-07	8.96E-05	~1%	CC	plasma membrane
4	GO:0016301	1.66E-04	2.65E-07	8.96E-05	~1%	MF	kinase activity
4	GO:0005737	3.66E-02	1.27E-04	2.24E-02	~1%	CC	cytoplasm
4	GO:0005515	1.74E-03	2.65E-07	8.96E-05	~0%	MF	protein binding
4	GO:0005524	1.85E-02	6.90E-06	1.83E-03	100%	MF	ATP binding
4	GO:0009736	3.28E-02	8.12E-05	1.77E-02	100%	BP	cytokinin mediated signaling pathway
4	GO:0042023	3.51E-02	1.08E-04	2.23E-02	100%	BP	DNA endoreduplication
4	GO:0009751	4.01E-02	1.83E-04	2.96E-02	50%	BP	response to salicylic acid stimulus
5	GO:0006270	2.72E-02	3.71E-05	1.54E-02	~95%	BP	DNA replication initiation
5	GO:0003700	2.14E-03	2.65E-07	3.29E-04	~83%	MF	transcription factor activity

5	GO:0009570	1.21E-02	2.39E-06	2.22E-03	~67%	CC	chloroplast stroma
5	GO:0009535	1.78E-02	8.49E-06	4.52E-03	~66%	CC	chloroplast thylakoid membrane
5	GO:0009941	3.29E-02	8.70E-05	3.14E-02	~65%	CC	chloroplast envelope
5	GO:0003677	2.09E-02	1.38E-05	6.42E-03	~3%	MF	DNA binding
5	GO:0005634	6.27E-03	2.65E-07	3.29E-04	~2%	CC	nucleus
5	GO:0003723	3.34E-02	9.28E-05	3.14E-02	~2%	MF	RNA binding
5	GO:0005739	1.53E-02	4.77E-06	2.96E-03	~12%	CC	mitochondrion
5	GO:0005515	3.53E-02	1.12E-04	3.49E-02	~0%	MF	protein binding
5	GO:0005524	1.47E-02	4.77E-06	2.96E-03	100%	MF	ATP binding
5	GO:0009507	2.09E-09	2.65E-07	3.29E-04	20%	CC	chloroplast
6	GO:0003700	3.42E-04	2.65E-07	3.33E-04	~83%	MF	transcription factor activity
6	GO:0003677	1.14E-02	2.65E-07	3.33E-04	~3%	MF	DNA binding
6	GO:0005634	1.30E-02	1.59E-06	1.50E-03	~2%	CC	nucleus
6	GO:0005886	5.77E-03	2.65E-07	3.33E-04	~1%	CC	plasma membrane
7	GO:0009941	2.27E-02	1.33E-05	1.66E-02	~65%	CC	chloroplast envelope
7	GO:0006839	2.94E-02	4.54E-05	3.41E-02	~28%	BP	mitochondrial transport
7	GO:0006412	3.13E-02	6.18E-05	3.87E-02	~20%	BP	translation
7	GO:0005739	1.71E-02	4.77E-06	8.97E-03	~12%	CC	mitochondrion
7	GO:0003735	2.56E-02	2.41E-05	2.27E-02	100%	MF	structural constituent of ribosome
7	GO:0009507	6.99E-03	2.65E-07	9.97E-04	20%	CC	chloroplast
