# Supplementary material for: Sex biased expression of hormone related genes at early stage of sex differentiation in papaya flowers
Source: Hortic Res. 2021 Jul 1;8:147. doi: 10.1038/s41438-021-00581-4 (PMC8245580; doi:10.1038/s41438-021-00581-4)
Supplement: Supplementary file 9 — Supplemental file 10 [file 41438_2021_581_MOESM9_ESM.pdf]

List of DEGs that located on the sex determination regions of papaya.

Gene ID	Annotation	M0 (FPKM)	F0 (FPKM)	log2(fold change)	Gene ID locus
CpXY1_MSY	Regulator of Vps4 activity in the MVB pathway	8.44	0.11	6.2177	MALE_Y_FINAL:3896142-3904027
CpXY10_X	Flowering locus t	3.42	0.7	2.286	CHRX-PSEUDOMOLECULE:3031985-3034259
CpXY11_X	PWWP domain-containing protein	9.94	31.45	-1.6619	CHRX-PSEUDOMOLECULE:3496542-3500489
CpXY12_MSY	ADP-ribosylation factor A1B	5.92	0.14	5.4154	MALE_Y_FINAL:1109954-1119373
CpXY12_X	ADP-ribosylation factor A1B	42.99	65.93	-0.6169	CHRX-PSEUDOMOLECULE:3679544-3689251
CpXY14_MSY	Monodehydroascorbate reductase	5.31	0	NA	MALE_Y_FINAL:866445-884414
CpXY14_X	Monodehydroascorbate reductase	7.34	13.01	-0.8259	CHRX-PSEUDOMOLECULE:3944239-3951345
CpXY16_MSY	Proteasome complex subunit	21.73	0	NA	MALE_Y_FINAL:4772320-4776903
CpXY16_X	Proteasome complex subunit	24.27	74.37	-1.6154	supercontig_1914:7049-11556
CpXY2_MSY	Somatic embryogenesis receptor kinase	12.56	0.05	7.9975	MALE_Y_FINAL:3888614-3894917
CpXY2_X	Somatic embryogenesis receptor kinase	17.54	29.88	-0.7684	supercontig_66:1185705-1191996
CpXY21_X	Porin family 3	1.09	1.11	-0.0181	supercontig_215:162271-177670
CpXY25_MSY	Hypothetical protein	2.43	0	NA	MALE_Y_FINAL:4975926-4976748
CpXY25_X	Hypothetical protein	2.37	3.07	-0.3734	supercontig_215:278304-278991
CpXY26_X	Phospholipid-transporting ATPase	2.81	4	-0.51	CHRX-PSEUDOMOLECULE:4438628-4469252
CpXY28_MSY	Proteasome assembly chaperone 3	4.04	0	NA	MALE_Y_FINAL:5122683-5139632
CpXY28_X	Proteasome assembly chaperone 3	13.79	19.25	-0.4815	CHRX-PSEUDOMOLECULE:4556989-4572019
CpXY29_X	N-acetylglucosamine-phosphate mutase	12.04	24.2	-1.0069	supercontig_39:2172070-2204341
CpXY3_MSY	Exocyst complex subunit SEC6	5.27	0.01	9.6139	MALE_Y_FINAL:3794114-3834089
CpXY3_X	Exocyst complex subunit SEC6	4.97	10.44	-1.0719	CHRX-PSEUDOMOLECULE:2048110-2091589
CpXY31_X	Asymmetric leaves 2	0.74	2.38	-1.6845	CHRX-PSEUDOMOLECULE:4737405-4740057
CpXY35_X	Calcium homeostasis regulater-like protein	3.24	4.05	-0.3215	CHRX-PSEUDOMOLECULE:4946388-4948081
CpXY36_X	Formate dehydrogenase	150.81	135.17	0.158	CHRX-PSEUDOMOLECULE:4948528-4951174
CpXY48_X	Mitochondrial NADH ubiquinone oxidoreductase 13kdlike subunit	0	28.61	NA	CHRX-PSEUDOMOLECULE:5323584-5330574
CpXY5_MSY	4-nitrophenylphosphatase	15.77	0.1	7.2997	MALE_Y_FINAL:1818802-1923480
CpXY5_X	4-nitrophenylphosphatase	21.98	38.76	-0.8184	CHRX-PSEUDOMOLECULE:2276560-2299372
CpXY50_X	Hypothetical protein	1.6	0	NA	CHRX-PSEUDOMOLECULE:5345038-5348590
CpXY6_MSY	FRA3 inositol or phosphatidylinositol phosphatase	1.37	0	16.3807	MALE_Y_FINAL:4435501-4505230
CpXY7_MSY	Leucine zipper-ef-hand (Ca binding motif)	2.57	0.01	7.964	MALE_Y_FINAL:4374968-4400948
CpXY7_X	Leucine zipper-ef-hand (Ca binding motif)	0.84	5.03	-2.5842	CHRX-PSEUDOMOLECULE:2654526-2674365
CpXY8_MSY	SYN4 (SISTER CHROMATID COHESION 1 PROTEIN 4	3.83	0	16.8195	MALE_Y_FINAL:1151879-1165918
CpXY8_X	SYN4 (SISTER CHROMATID COHESION 1 PROTEIN 4	2.55	6.57	-1.3631	CHRX-PSEUDOMOLECULE:2698976-2713081

CpXY9_MSY	Transcription regulator	21.04	0.09	7.9468	MALE_Y_FINAL:1206614-1209779
CpXY9_X	Transcription regulator	38.48	99.6	-1.3722	CHRX-PSEUDOMOLECULE:2788980-2792337
PXCpXY22_MS	---NA---	2.55	0.02	6.6939	MALE_Y_FINAL:7516896-7542415
PXCpXY22_X	---NA---	9.07	12.15	-0.4225	CHRX-PSEUDOMOLECULE:3385130-3423902
PXCpXY6_MSY	latex cyanogenic beta glucosidase	14.42	4.99	1.5307	MALE_Y_FINAL:1234923-1300642
PXYCpXY1_X	universal stress protein (USP) family protein	16.28	29.59	-0.8622	CHRX-PSEUDOMOLECULE:3455557-3493735
PXYCpXY3_M	chloroplast ribosomal protein L12 and tRNA-Protein	31.52	0.19	7.3735	MALE_Y_FINAL:4290899-4291615
PXYCpXY3_X	chloroplast ribosomal protein L12 and tRNA-Protein	19.74	32.49	-0.7189	supercontig_39:1974216-1979243
PXYCpXY4_M	---NA---	0.54	1.95	-1.8365	MALE_Y_FINAL:8012349-8019502
CpY-16_MSY	---NA---	1.27	0	22.1968	MALE_Y_FINAL:2106915-2128652
CpY-19_MSY	---NA---	43.43	0.07	9.1973	MALE_Y_FINAL:3981068-4004433
CpY-1-msycopy	---NA---	11.71	0	NA	MALE_Y_FINAL:28562-111103
CpY-20_MSY	---NA---	7.3	0	NA	MALE_Y_FINAL:3981068-4004433
CpY-8_MSY	---NA---	2.34	0.01	7.6747	MALE_Y_FINAL:1234923-1300642
PCpY-1_MSY	---NA---	9.09	0	23.7639	MALE_Y_FINAL:477330-479081
PCpY-9_MSY	---NA---	1.65	0.15	3.4513	MALE_Y_FINAL:2106915-2128652
PYCpXY5_MSY	---NA---	17.71	0	NA	MALE_Y_FINAL:3712421-3713594
PYCpXY22_X	---NA---	1.66	0.96	0.779	CHRX-PSEUDOMOLECULE:3385130-3423902
PYCpXY23_X	---NA---	0	1.13	NA	CHRX-PSEUDOMOLECULE:3455557-3493735
PYCpXY28_X_	---NA---	0.51	1.17	-1.1848	CHRX-PSEUDOMOLECULE:4338024-4346526
PYCpXY30_X	---NA---	0.75	1.07	-0.51	CHRX-PSEUDOMOLECULE:4533020-4546847
PYCpXY5_X	---NA---	5.23	8.6	-0.7178	CHRX-PSEUDOMOLECULE:2130442-2135901
PYCpXY7_X	---NA---	10.22	14.29	-0.4838	CHRX-PSEUDOMOLECULE:2253157-2263261
CpX-1	---NA---	5.38	10.39	-0.9494	CHRX-PSEUDOMOLECULE:1952688-1957129
CpX-15	---NA---	5.31	7.66	-0.5277	CHRX-PSEUDOMOLECULE:2371022-2371797

NA: Not Available
