# Supplementary material for: Sex biased expression of hormone related genes at early stage of sex differentiation in papaya flowers
Source: Hortic Res. 2021 Jul 1;8:147. doi: 10.1038/s41438-021-00581-4 (PMC8245580; doi:10.1038/s41438-021-00581-4)
Supplement: Supplementary file 10 — Supplemental file 11 [file 41438_2021_581_MOESM10_ESM.pdf]

Primers sequences used for qRT-PCR in this study.

Gene	forward symbol	forward primer sequence	reverse symbol	reverse primer sequence	Amplicon length (bp)	Amplicon Tm (℃)
evm.TU.contig_32826.1	*C32826.1-F*	GAGCTCGTTGTCACCACCTATGCT	*C32826.1-R*	CCTTGTTCTTGAATCCAGCCACTC	82	84.1
CpXYh11_X	*CpXYh11_X-F*	TACAGAAGGCAGTTGATGAGATG	*CpXYh11_X-R*	TTTGGTGGGTTGAAATAAAGATT	185	84.8
CpXYh2_X	*CpXYh2_X-F*	CTGGTGCTGCCTTGCTGTTTGCT	*CpXYh2_X-R*	TCTGTTGCGACTTGTAATTCCCG	163	86.2
CpXYh8_X	*CpXYh8_X -F*	GATGATACAGCGGCAGTTCTTAG	*CpXYh8_X -R*	TTTTTTCAACAACATCAAGCCTA	200	83.9
CpY-19_MSY	*CpY-19_MSY -F*	CTGGAACAGCCATCTCTTGAACT	*CpY-19_MSY -R*	TTGCTCTATGCTTAATCCTTGGA	135	85.8
evm.TU.supercontig_131.87	*S131.87-F*	CTTATTCGTCCTTTCACACTACTC	*S131.87-R*	GATGACAATTTGTATGGAAGATCA	160	84.6
evm.TU.supercontig_1346.4	*S1346.4-F*	CCCAGAACTGTCACCAAGAATCAC	*S1346.4-R*	CATCCATGCTCACTTTCACATAGA	153	87
evm.TU.supercontig_292.1	*S292.1-F*	AGTTGCTCTACAGTCTGCTAATGC	*S292.1-R*	GGGTCTTGAAATGGTCACTCTTAT	168	86.4
evm.TU.supercontig_3.199	*S3.199-F*	CTCTCATCATCTTTTCTAACCGTG	*S3.199-R*	CCTTGTGCCCCATAACTACATTGA	106	81.4
evm.TU.supercontig_36.134	*S36.134-F*	GAGAAGCCTCGGTACTCATCTCAT	*S36.134-R*	GTGCAGTTAACACCTCCTTTGCCC	164	86.4
evm.TU.supercontig_376.4	*S376.4-F*	CAGAGACCCAACCAACTCCGACA	*S376.4-R*	TGTATCGGCTAATGAGAGACCAA	119	87.8
evm.TU.supercontig_38.71	*S38.71-F*	GCTAACCAAAACCCAGTTGATGT	*S38.71-R*	ATAGATGTGGAGGAAGAGGTTGA	163	84
evm.TU.supercontig_414.3	*S414.3-F*	ATAGCCAAAACCCTGGAGCGATAC	*S414.3-R*	TTTTGCCTTCAACTTTGCTACCTC	114	84.9
evm.TU.supercontig_471.4	*S471.4-F*	TTAAGCGTGAAGGAGCTAAAACAA	*S471.4-R*	TCCACTTCTGCTATCTTAGTCCGA	170	85
evm.TU.supercontig_49.92	*S49.92-F*	AGGCAGCACCACCACCTCCTCAG	*S49.92-R*	GTTGTGCCCATCGCCTGTGTTAC	160	86.9
evm.TU.supercontig_58.29	*S58.29-F*	CTCTGAGACTGTTGATTTGAAGC	*S58.29-R*	CTCTTCTGAGCCATTATGTTCTT	198	85.7
evm.TU.supercontig_59.41	*S59.41-F*	GGATACACGGAACTGCTGGAGAT	*S59.41-R*	CCACAACCCACTTTGGTAAAGGA	127	87.7
evm.TU.supercontig_78.79	*S78.79-F*	TCCTTGGCTTGTCGTCAGAGATT	*S78.79-R*	GAGGCGGGAGAGAATGAGCAACA	142	87.5
Polyubiquitin	*UBQ-F*	CCTTCTATATGAATGCCTAGC	*UBQ-R*	CAGGACATACCAATATCACA	143	76.5
