# Supplementary figures and images for: Sex biased expression of hormone related genes at early stage of sex differentiation in papaya flowers
Source: Hortic Res. 2021 Jul 1;8:147. doi: 10.1038/s41438-021-00581-4 (PMC8245580; doi:10.1038/s41438-021-00581-4)

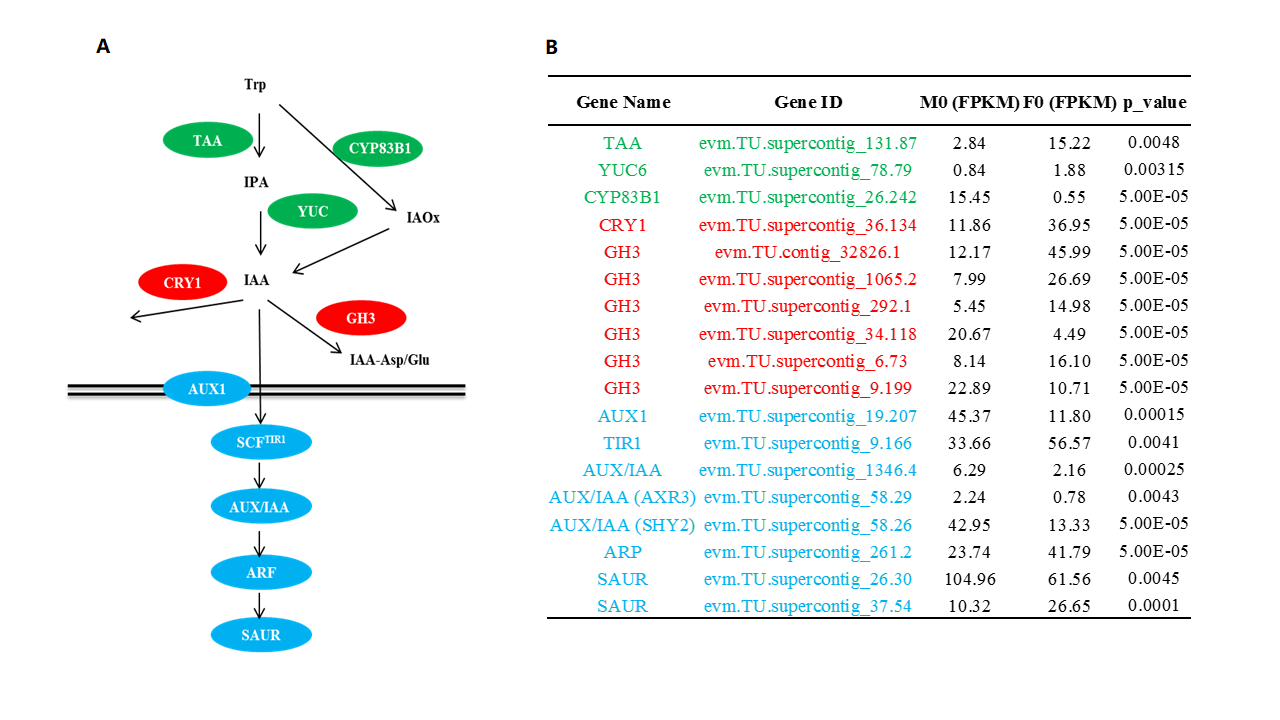

Supplement: Supplementary file 11 — Supplemental file 6 [file 41438_2021_581_MOESM11_ESM.tif]
