# Supplementary material for: Sex biased expression of hormone related genes at early stage of sex differentiation in papaya flowers
Source: Hortic Res. 2021 Jul 1;8:147. doi: 10.1038/s41438-021-00581-4 (PMC8245580; doi:10.1038/s41438-021-00581-4)
Supplement: Supplementary file 11 [file 41438_2021_581_MOESM1_ESM.docx]

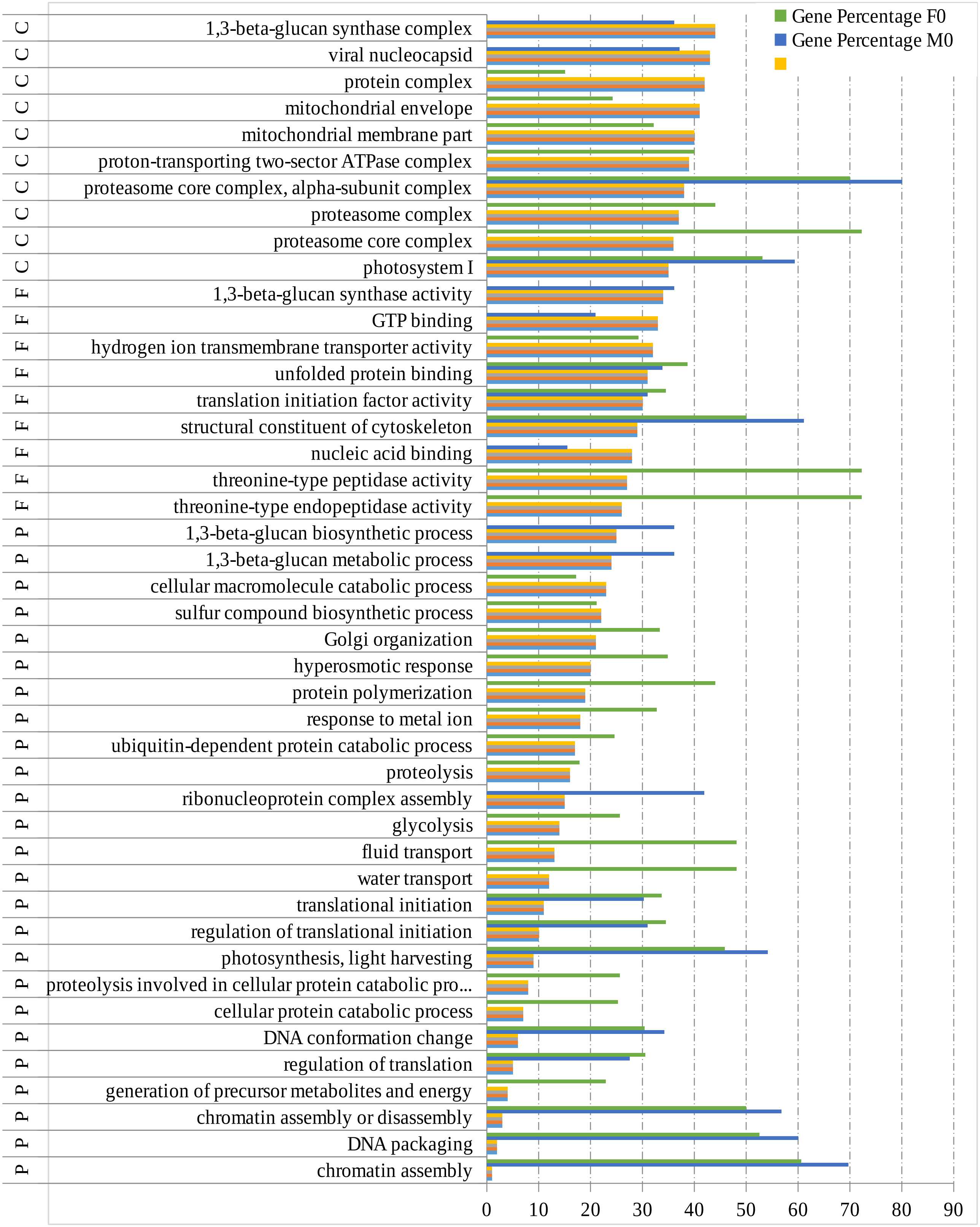

Gene Percentage F0
Gene Percentage M0
C 1,3-beta-glucan synthase complex
C viral nucleocapsid
C protein complex
C mitochondrial envelope
C mitochondrial membrane part
C proton-transporting two-sector ATPase complex
C proteasome core complex, alpha-subunit complex
C proteasome complex
C proteasome core complex
C photosystem I
F 1,3-beta-glucan synthase activity
F GTP binding
F hydrogen ion transmembrane transporter activity
F unfolded protein binding
F translation initiation factor activity
F structural constituent of cytoskeleton
F nucleic acid binding
F threonine-type peptidase activity
F threonine-type endopeptidase activity
P 1,3-beta-glucan biosynthetic process
P 1,3-beta-glucan metabolic process
P cellular macromolecule catabolic process
P sulfur compound biosynthetic process
P Golgi organization
P hyperosmotic response
P protein polymerization
P response to metal ion
P ubiquitin-dependent protein catabolic process
P proteolysis
P ribonucleoprotein complex assembly
P glycolysis
P fluid transport
P water transport
P translational initiation
P regulation of translational initiation
P photosynthesis, light harvesting
P proteolysis involved in cellular protein catabolic pro...
P cellular protein catabolic process
P DNA conformation change
P regulation of translation
P generation of precursor metabolites and energy
P chromatin assembly or disassembly
P DNA packaging
P chromatin assembly
0 10 20 30 40 50 60 70 80 90
